# Differentiation into an Effector Memory Phenotype Potentiates HIV-1 Latency Reversal in CD4^+^ T Cells

**DOI:** 10.1128/JVI.00969-19

**Published:** 2019-11-26

**Authors:** Deanna A. Kulpa, Aarthi Talla, Jessica H. Brehm, Susan Pereira Ribeiro, Sally Yuan, Anne-Gaelle Bebin-Blackwell, Michael Miller, Richard Barnard, Steven G. Deeks, Daria Hazuda, Nicolas Chomont, Rafick-Pierre Sékaly

**Affiliations:** a Department of Pediatrics, Emory University, Atlanta, Georgia, USA; b Department of Pathology, Case Western Reserve University, Cleveland, Ohio, USA; c ViiV Healthcare, Research Triangle Park, North Carolina, USA; d Janssen Pharmaceuticals, Springhouse, Pennsylvania, USA; e University of Georgia, Athens, Georgia, USA; f Infectious Disease, Merck & Co., Inc., West Point, Pennsylvania, USA; g Department of Medicine, University of California, San Francisco, San Francisco, California, USA; h Centre de Recherche du CHUM, Montréal, Quebec, Canada; i Université de Montréal, Department of Microbiology, Infectiology, and Immunology, Montréal, Quebec, Canada; Icahn School of Medicine at Mount Sinai

**Keywords:** CD4 T cells, HIV latency, HIV persistence

## Abstract

During antiretroviral therapy (ART), human immunodeficiency virus type 1 (HIV-1) persists as a latent reservoir in CD4^+^ T cell subsets in central memory (T_CM_), transitional memory (T_TM_), and effector memory (T_EM_) CD4^+^ T cells. We have identified differences in mechanisms underlying latency and responses to latency-reversing agents (LRAs) in *ex vivo* CD4^+^ memory T cells from virally suppressed HIV-infected individuals and in an *in vitro* primary cell model of HIV-1 latency. Our *ex vivo* and *in vitro* results demonstrate the association of transcriptional pathways of T cell differentiation, acquisition of effector function, and cell cycle entry in response to LRAs. Analyses of memory cell subsets showed that effector memory pathways and cell surface markers of activation and proliferation in the T_EM_ subset are predictive of higher frequencies of cells carrying an inducible reservoir. Transcriptional profiling also demonstrated that the epigenetic machinery (known to control latency and reactivation) in the T_EM_ subset is associated with frequencies of cells with HIV-integrated DNA and inducible HIV multispliced RNA. T_CM_ cells were triggered to differentiate into T_EM_ cells when they were exposed to LRAs, and this increase of T_EM_ subset frequencies upon LRA stimulation was positively associated with higher numbers of p24^+^ cells. Together, these data highlight differences in underlying biological latency control in different memory CD4^+^ T cell subsets which harbor latent HIV *in vivo* and support a role for differentiation into a T_EM_ phenotype in facilitating latency reversal.

**IMPORTANCE** By performing phenotypic analysis of latency reversal in CD4^+^ T cells from virally suppressed individuals, we identify the T_EM_ subset as the largest contributor to the inducible HIV reservoir. Differential responses of memory CD4^+^ T cell subsets to latency-reversing agents (LRAs) demonstrate that HIV gene expression is associated with heightened expression of transcriptional pathways associated with differentiation, acquisition of effector function, and cell cycle entry. *In vitro* modeling of the latent HIV reservoir in memory CD4^+^ T cell subsets identify LRAs that reverse latency with ranges of efficiency and specificity. We found that therapeutic induction of latency reversal is associated with upregulation of identical sets of T_EM_-associated genes and cell surface markers shown to be associated with latency reversal in our *ex vivo* and *in vitro* models. Together, these data support the idea that the effector memory phenotype supports HIV latency reversal in CD4^+^ T cells.

## Abstract (precis)

By performing phenotypic analysis of latency reversal in CD4^+^ T cells from virally suppressed individuals, we identify the T_EM_ subset as the largest contributor to the inducible HIV reservoir. Differential responses of memory CD4^+^ T cell subsets to latency-reversing agents (LRAs) demonstrate that HIV gene expression is associated with heightened expression of transcriptional pathways associated with differentiation, acquisition of effector function, and cell cycle entry. *In vitro* modeling of the latent HIV reservoir in memory CD4^+^ T cell subsets identify LRAs that reverse latency with ranges of efficiency and specificity. We found that therapeutic induction of latency reversal is associated with upregulation of identical sets of T_EM_-associated genes and cell surface markers shown to be associated with latency reversal in our *ex vivo* and *in vitro* models. Together, these data support the idea that the effector memory phenotype supports HIV latency reversal in CD4^+^ T cells.

## INTRODUCTION

The lifelong persistence of latently infected cells is a major obstacle toward human immunodeficiency virus type 1 (HIV-1) eradication in individuals on long-term antiretroviral therapy (ART). HIV-1 persists in a variety of different cells, including naive CD4^+^ T cells and myeloid cells; however, the majority of proviral HIV-1 DNA is found in CD4^+^ T cells displaying a memory phenotype (1–6). Memory CD4^+^ T cells, such as central memory (T_CM_), transitional memory (T_TM_), and effector memory (T_EM_) CD4^+^ T cells, are each endowed with very distinct functional and survival properties; T_EM_ cells rapidly upregulate effector function, and T_CM_ cells preserve immune memory by upregulating pathways that aid in long-term (years to decades) survival (3, 4). T_CM_ cells express the costimulatory molecule CD27 (7) as well as CD62L and CCR7, two surface molecules that facilitate migration to T cell zones within lymph nodes and mucosal lymphoid organs. T_CM_ cells are characterized by a delay in effector cytokine production (gamma interferon [IFN-γ], tumor necrosis factor alpha [TNF-α]) after T cell receptor (TCR) stimulation; however, they readily proliferate and differentiate into effector cells in response to antigenic stimulation (reviewed in reference 8). T_CM_ cells are more resistant to Fas-induced apoptosis than T_EM_ cells (9–12); their apoptosis is mediated through the activation and phosphorylation of STAT5a and inactivation of the transcriptional repressor FOXO3a (13). In contrast, T_EM_ cells (CCR7^−^ CD27^−^) express homing receptors for migration to nonlymphoid sites of inflammation and have high levels of gut-homing molecules (α_4_β_7_ integrin) and chemokine receptors that target these cells to nonlymphoid tissues (reviewed in reference 8). T_CM_ and T_EM_ cells are maintained through homeostatic proliferation, although T_EM_ cells show more rapid turnover, indicating that these cells are replaced at a higher rate (14). T_TM_ cells (CCR7^–^ CD27^+^) display an intermediate phenotype characterized by lower responsiveness to interleukin 15 (IL-15) than that of T_EM_ cells and transcript expression levels that more closely align with T_EM_ cells for some transcripts (*CD62L*, *TOSO*, and *PIM2*) and with T_CM_ cells for others (*Bim*, *FasL*, and *IFN-γ* [13, 15–18]). T_CM_ and T_EM_ cells show distinct epigenetic profiles, as T_EM_ cells are poised to respond to antigen and quickly produce effector cytokines, whereas T_CM_ cells are quiescent cells that require strong stimulation and costimulation to respond to their cognate antigen (19, 20). Significantly, all memory CD4^+^ T cell subsets have been shown to contribute to HIV persistence and harbor replication-competent HIV-1 (1, 3–5, 21–23), but recent evidence has suggested that T_EM_ cells harbor more intact HIV-1 provirus than either T_CM_ or T_TM_ cells (24). However, the mechanisms responsible for the persistence of the HIV-1 reservoir in these distinct memory CD4^+^ T cell subsets *in vivo* are still largely unknown, which may be critical for the development of effective eradication strategies.

One eradication strategy, the “shock and kill” approach, aims to eliminate the HIV-1 reservoir through latency reversal and immunological clearance (25). Given the inherent molecular differences that define the biology of T_CM_, T_TM_, and T_EM_ cells, it is unclear whether the same interventions will be equally effective in these distinct populations. Theoretically, the different activation states and basal expression levels of transcription factors within these subsets might affect the activity of latency-reversing agents (LRAs). Here, we examine the impact of the memory CD4^+^ T cell subset phenotype on HIV-1 latency reversal. We show in *ex vivo* and *in vitro* models that the differentiated phenotype of T_EM_ cells from that of quiescent T_CM_ cells is associated with a brisker response to LRAs, suggesting that differentiation of latently infected cells into T_EM_ cells may facilitate their elimination in the context of a shock and kill approach.

## RESULTS

### The T_EM_ subset shows the highest levels of the inducible HIV reservoir.

**FIG 1:**
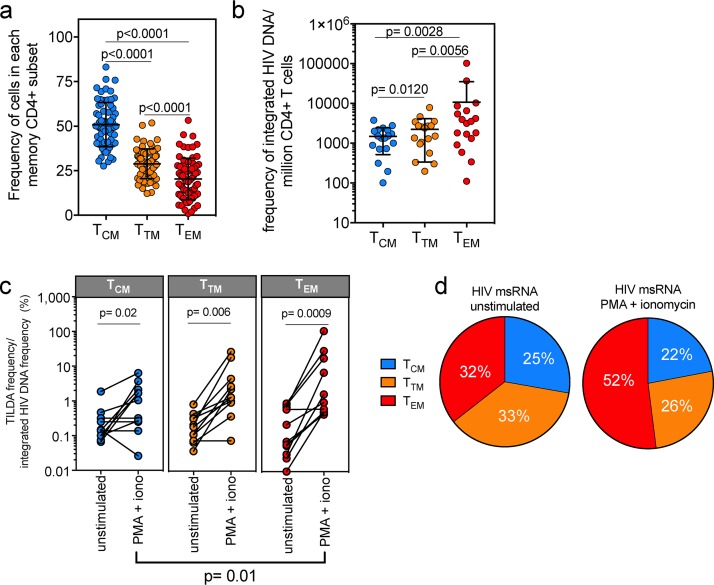
The inducible HIV reservoir resides in the T_EM_ subset. (a) The distribution of the T_CM_, T_TM_, and T_EM_ subsets in *ex vivo* memory CD4^+^ T cells is shown, and *P* values are indicated. Each circle represents individual participants from Table S1 in the supplemental material (bars indicate means with standard deviations [SD], Wilcoxon matched-pair signed-rank test, *n* = 69). (b) CD4^+^ T cells from virally suppressed individuals were sorted into T_CM_, T_TM_, and T_EM_ subsets using the gating strategy in Fig. S1 in the supplemental material, and integrated HIV DNA in each subset was quantified. Bars indicate means with SD from a Wilcoxon matched-pair signed-rank test; *P* values are indicated (*n* = 18). (c) The frequency of transcription of HIV msRNA induced by PMA plus ionomycin (iono), normalized by the amount of integrated HIV DNA in each subset, is shown. Unstimulated sorted subsets were included as a control. There was no statistical difference between levels of HIV msRNA in cells from the CD4^+^ memory subsets before activation. After exposure to PMA and ionomycin, the frequency of HIV msRNA was quantified and calculated. *P* values are indicated. (Wilcoxon matched-pair signed-rank test; *n* = 11). (d) Contribution of HIV msRNA from each CD4^+^ memory subset from panel c. All undetectable values were input as zero. The contributions of the T_CM_, T_TM_, and T_EM_ subsets to the overall HIV msRNA signal are shown. Each pie slice was calculated using the frequency of HIV msRNA in each memory subset as a percentage of the total signal. Frequency is indicated within each pie slice (*n* = 11).

The diversity of transcriptional and functional programs of memory CD4^+^ T subsets (3, 4, 7–14) led us to hypothesize that the subsets show varied capacities to support HIV-1 latency reversal. We characterized T_CM_, T_TM_, and T_EM_ cells from two cohorts of virally suppressed individuals, one from San Francisco, CA (Study of the Consequences of the Protease Inhibitor Era [SCOPE] cohort; *n* = 47), and the other from Fort Pierce, FL (FL cohort; *n* = 22) (see Table S1 in the supplemental material). From peripheral blood mononuclear cells (PBMCs) from these two cohorts, we measured the memory cell subset frequencies in each participant and identified the T_CM_ subset to be the most prevalent in memory (CD45RA^−^) CD4^+^ T cells (mean, 51%; *P* < 0.0001) (Fig. 1a; Fig. S1a) compared to the T_TM_ and T_EM_ subsets (means, 29% and 20%, respectively). We obtained apheresis products from 18 participants from the FL cohort in order to sort *ex vivo* T_CM_, T_TM_, and T_EM_ cells and then identified the T_EM_ subset to have the highest frequency of integrated HIV DNA (Fig. 1b) (26). After normalization to the proportion of cells in each subset, there was no difference in the contributions of the subsets to the reservoir (Fig. S1b). To determine the size of the inducible reservoir in each memory CD4^+^ T cell subset, we used the *Tat*/*rev*-induced limiting dilution assay (TILDA), which quantifies the frequency of cells induced to express HIV multiply spliced RNA (HIV msRNA) after phorbol myristate acetate (PMA) plus ionomycin treatment (27). Eleven participants from the FL cohort were then selected to represent the range of integrated HIV DNA frequencies that we found within in each subset (Fig. S1c), and TILDA was performed on sorted T_CM_, T_TM_, and T_EM_ cell populations (Fig. 1c). After normalization of inducible HIV msRNA expression to the frequency of integrated HIV DNA within each subset, we determined that the T_EM_ subset encompassed higher frequencies of cells with inducible genomes than the T_CM_ subset (*P* = 0.01; median for T_EM_ cells = 22-fold, median for T_TM_ cells = 8-fold, median for T_CM_ cells = 3-fold) (Fig. 1c) and also was the largest contributor to the pool of cells producing HIV msRNA (Fig. 1d). These data show that although all memory CD4^+^ T cell subsets contribute to HIV persistence (1), the T_EM_ subset shows the highest frequency of cells with an inducible reservoir.

### Effector T cell differentiation and cell cycle entry transcriptional programs are associated with HIV reactivation.

**FIG 2:**
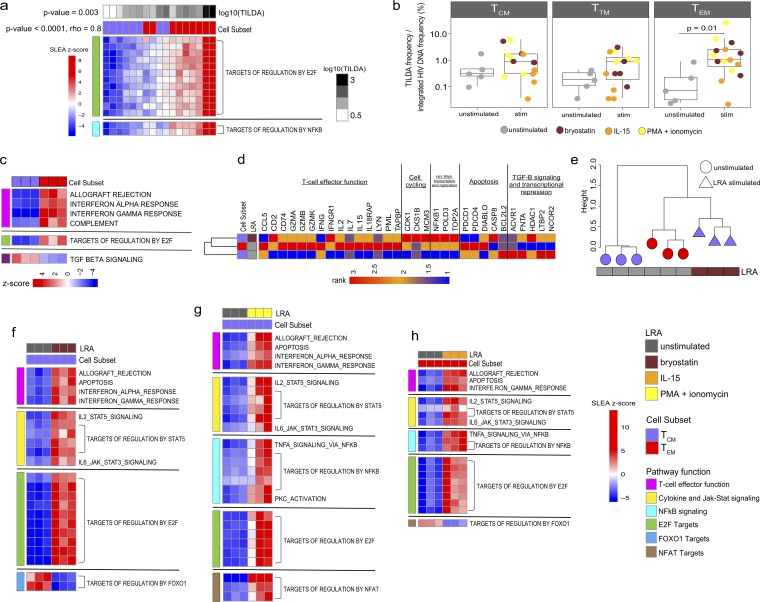
Latency reversal is associated with the upregulation of T cell effector pathways. (a) Heatmap of the gene sets expressed in sorted *ex vivo* T_EM_ and T_CM_ cells from the FL cohort correlated with the frequency of cells expressing HIV msRNA measured by TILDA (*n* = 9). GSEA was first performed to identify pathways/gene sets correlated with TILDA across the cell subsets, and then the SLEA z-score of the pathway was calculated per sample, represented by the color gradient. Rows represent the pathway, and columns represent samples. Samples were ordered by increasing expression of the pathway associated with TILDA (mean rank ordering). The association between the mean rank and TILDA is then tested with a Spearman correlation, and its *P* value and rho are indicated next to the TILDA annotation bar. The increasing pathway expression is associated with increasing TILDA values (*P* value < 0.0001, rho = 0.8). A Wilcox test is then performed to assess the difference in mean ranks between T_EM_ and T_CM_ cells. The Wilcox test *P* value is then indicated next to the cell subset annotation bar. These gene sets also show higher expression in T_EM_ cells than in T_CM_ cells (*P* value = 0.003). (b) We examined the inducible reservoir in sorted memory CD4^+^ T cell subsets from virally suppressed HIV-infected individuals (FL cohort; *n* = 5). Sorted memory CD4^+^ T_CM_, T_TM_, and T_EM_ subsets were exposed to 100 ng/ml PMA plus 1 μg/ml ionomycin, 10 ng/ml IL-15, or 10 nM bryostatin, and the induction of HIV msRNA was measured using TILDA (stimulated [stim]). Untreated cells were used as a control (unstim). The T_EM_ and T_TM_ subsets showed significant induction of HIV msRNA upon stimulation compared to that of the unstimulated controls (Wilcoxon rank sum test; *P* values are indicated). The line in each box plot denotes a median value with SD. (c) Heatmap of the pathways differentially expressed between unstimulated (baseline) CD4^+^ memory subsets (*P* value < 0.05) from three out of the five individuals shown in panel b. Rows represent the pathway, and columns represent samples. The color gradient represents the z-score of the pathway per sample calculated by SLEA. Effector function and cell cycling pathways show significant upregulation in the T_EM_ subset compared to in the T_CM_ and T_TM_ subsets, while the senescent TGF-β signaling pathway is significantly upregulated in the T_CM_ subset. (d) Heatmap of the genes upregulated in the unstimulated T_EM_ subset compared to those of the unstimulated T_CM_ subset. The average gene expression across the three participants (shown in panel b) per memory subset is shown here. The expression of these genes was also assessed in the bryostatin-stimulated T_CM_ cells, showing that the stimulated T_CM_ cells have a gene expression profile similar to that of the unstimulated T_EM_ cells. The color gradient represents the rank of the gene expression across subsets (*n* = 3). (e) Hierarchical clustering on the pathways in the T_CM_ subset upon exposure to bryostatin that was differentially expressed from that of the unstimulated controls (*P* value < 0.05). The pathway expression was assessed in the T_CM_ subset and in the unstimulated T_EM_ subset. Similarities in gene expression were determined using the complete linkage clustering method, with the distance between gene expression profiles measured using Euclidean distance. The height of a dendrogram represents the Euclidean distance. The bryostatin-stimulated T_CM_ subset is in close proximity to the unstimulated T_EM_ subset. (f and g) Heatmap of the pathways in the T_CM_ subset upon exposure to LRAs (bryostatin [e] or PMA plus ionomycin [f]) that were differentially expressed from those of their unstimulated controls (*P* value < 0.05). Rows represent the pathway, and columns represent samples. The color gradient represents the z-scores of the pathway per sample calculated by SLEA. Pathways are grouped by biological function. (h) Heatmap of the pathways differentially expressed in the T_EM_ subset upon exposure to IL-15 (*P* value < 0.05).

Based on these data, we used a transcriptomics approach to test the hypothesis that a T_EM_ cell-specific gene signature supported HIV expression at a higher frequency than that of a T_CM_ cell. We initially assessed the correlation between pathways and HIV expression in *ex vivo*-sorted memory CD4^+^ T cell subsets from nine FL cohort participants that represented the range of TILDA frequencies measured in Fig. 1c (T_CM_ cell range in TILDA values, 1.1 to 85; T_EM_ cell range in TILDA values, 5.6 to 2,506). Results illustrated in Fig. 2a demonstrate that cell cycling expression (targets of E2F transcription factors) and targets of NF-κB expressed *ex vivo* significantly correlated with the frequency of the inducible reservoir in T_CM_ or T_EM_ cells (*P* value < 0.001, rho = 0.8) and that these pathways were preferentially expressed more highly in the T_EM_ cells (*P* value = 0.003), highlighting the importance of proliferation and NF-κB (known to enhance viral replication) in the capacity to respond to PMA plus ionomycin activation. Memory CD4^+^ T cell subsets from five participants from the FL cohort were further assessed for responses to LRAs. We quantified HIV msRNA from sorted memory subsets that had been exposed to three distinct LRAs, the protein kinase C (PKC) agonist bryostatin, the γ-chain cytokine IL-15, or PMA plus ionomycin. T_EM_ cells again showed significant induction of HIV msRNA expression, with a mean 35-fold increase (*P* = 0.01) (Fig. 2b) compared to its expression in other subsets. Transcriptional profiling was then used to identify mechanisms specific to T_CM_, T_TM_, and T_EM_ cells associated with LRA responses in three out of the five participants. First, we confirmed that T_EM_ cells intrinsically (prestimulation) showed higher expression of pathways and genes associated with effector function, cell cycle progression, and HIV RNA transcription and replication than T_CM_ cells (Fig. 2c; Table S2) (28). Here, the *ex vivo* T_CM_ cells showed higher expression of quiescence signatures, such as epigenetic repressors (HDAC1 and NCOR2) and genes downstream of the TGF-β signaling pathway (ACVR1, FNTA, LTBP2) (Fig. 2c, Table S2) (29). However, after bryostatin stimulation, the T_CM_ subset was induced to express genes involved in effector function, cell cycling, and HIV transcription, indicating that the exposure of T_CM_ cells to LRAs triggered the expression of pathways and genes which are hallmarks of T_EM_ cells (Fig. 2d). Hierarchical clustering of these pathways showed that the bryostatin-stimulated T_CM_ subset exhibited pathway expression profiles similar to those of the unstimulated T_EM_ subset (Fig. 2e). Transcriptional profiles of the T_CM_ subset exposed to PMA plus ionomycin activation also showed gene and pathway similarity to those of unstimulated T_EM_ cells (Fig. S2a).

The pathways modulated in T_CM_ cells upon exposure to bryostatin or PMA plus ionomycin included genes encoding several cytokines with effector functions (gamma interferon [IFN-γ], IL-2, IL-9, tumor necrosis factor alpha [TNF-α], granzyme A [GZMA]) as well as activation markers (CD38, CD69, CD25 IL2RA) (Fig. 2f and g; Table S3). Importantly, T_CM_ cells upregulated the expression of transcription factors known to activate HIV-1 transcription; these factors included NFAT5, STAT5, NF-κB, E2F, and their targets, i.e., topoisomerase 2A (TOP2A) (30–32) and the eukaryotic translation apparatus (EIF2S1, EIF4A1) (33). The PKC activation pathway, known to facilitate HIV-1 latency reversal by triggering NF-κB (34–36), was also upregulated in T_CM_ cells exposed to PMA plus ionomycin (Fig. 2g). These effector pathways also were all upregulated in T_EM_ cells upon exposure to LRAs, including the kinase PIM-1 (37), a major contributor to HIV-1 reactivation (Fig. S2b; Table S4).

Significantly, bryostatin downregulated FOXO1 and its targets (a transcriptional regulator that controls the differentiation of T_CM_ cells [38–41]) in T_CM_ cells (Fig. 2f). These included the FOXO1 gene and genes like the transcription factor that controls the maintenance of the memory T cell stem cell program TCF7, the cell cycle inhibitor CDKN1A, the chromatin-remodeling protein ARID4A, which recruits histone deacetylases (HDACs) to silence HIV-1, the transcriptional repressor MAFF, and the BCL2 family gene *BMF*. In T_EM_ cells, bryostatin-downregulated FOXO1 targets included RUNX1, known to bind the HIV long terminal repeat (LTR) and to repress transcription (42), and BMF (Fig. 2f; Fig. S2b; Table S3; Table S4). Both ID2 and IRF4, factors critical for promoting the differentiation of T_CM_ to T_EM_ cells, were upregulated in T_CM_ and T_EM_ cells upon exposure to bryostatin and/or PMA plus ionomycin (Table S3; Table S4) (43, 44; unpublished data). In addition, the upregulation of these effector pathways and downregulation of FOXO1 and its targets were also observed specifically in the T_EM_ subset upon stimulation with IL-15 (Fig. 2h). PMA plus ionomycin stimulation of T_TM_ cells also triggered the upregulation of the above-mentioned pathways, all of which overlapped those expressed by T_CM_ and T_EM_ cells (Fig. S2c; Table S3).

### Markers of T cell activation predict the inducible HIV reservoir in memory subsets.

**FIG 3:**
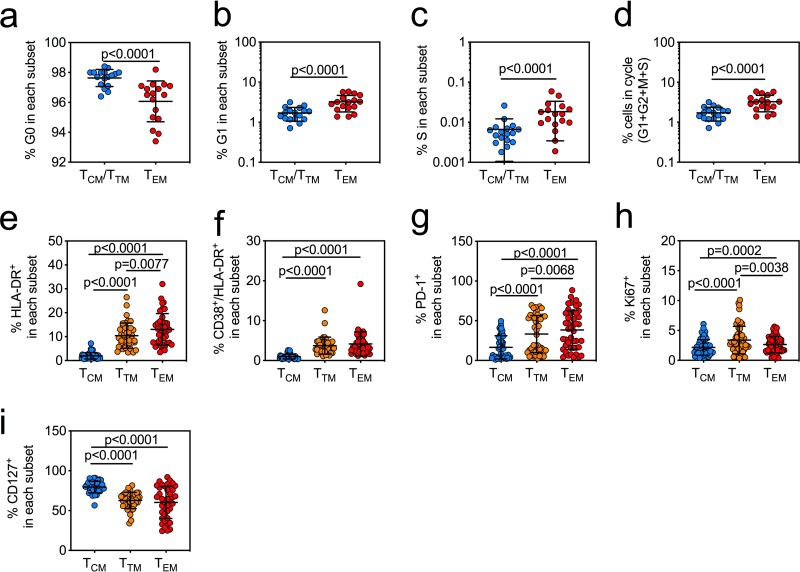
The T_EM_ subset is characterized by expression of markers of cell cycle entry and of T cell activation. (a to c) Percentages of CD4^+^ T cells in the T_CM_/T_TM_ subsets (blue circles) and T_EM_ subsets (red circles) in resting phase (G_0_) (a), growth phase (G_1_) (b), and DNA synthesis phase (S) and mitosis phase (M) (c) of the cell cycle from virally suppressed HIV-infected individuals (bars indicate means with SD from a Wilcoxon matched-pair signed-rank test [*n* = 17]). (d) Percentages of cells in the cycle are represented with the equation [(G_1_ + G_2_ + M + S)/(G_0_ + G_1_ + G_2_ + M + S)] × 100, which shows significantly more cycling cells in the T_EM_ subset than in other subsets (bars indicate means with SD from a Wilcoxon matched-pair signed-rank test [*n* = 17]). (e to i) Percentages of cells in the T_CM_ (blue circles), T_TM_ (orange circles), and T_EM_ (red circles) subsets positive for HLA-DR (*n* = 45) (e) or coexpressing CD38/HLA-DR (*n* = 45) (f), PD-1 (*n* = 45) (g), Ki67 (*n* = 57) (h), or CD127 (*n* = 40) (i). *P* values are indicated from a Mann-Whitney test. Measurements were based upon the availability of cells from each cohort.

Transcriptional profiling showed that LRAs triggered the expression of genes associated with T cell activation and cell cycle entry (Fig. 2c to h). To determine if expression of these pathways *ex vivo* correlated with the frequency of cells that harbor inducible HIV, we quantified T_CM_, T_TM_, and T_EM_ cells for cell cycle frequency and activation marker expression in cells from the SCOPE and FL cohorts from Fig. 1 (Fig. 3). We quantified the proportions of *ex vivo* CD4^+^ T cells from virally suppressed HIV-infected individuals in resting (G_0_) or active (G_1_, S, and G_2_M) cells and found that T_EM_ cells had significantly higher frequencies of cells progressing through the cell cycle (*P* < 0.0001) (Fig. 3a to d). T_TM_ and T_EM_ cells also had significantly higher frequencies of the activation and proliferation markers HLA-DR (*P* < 0.0001), CD38 (*P* < 0.0001), PD-1 (*P* < 0.0001), and Ki67 (*P* < 0.0001, *P* = 0.0002), while T_CM_ cells maintained the highest levels of CD127 (IL-7Rα) (*P* < 0.0001) (Fig. 3e to i). CD127 expression is associated with a greater ability to become a long-term memory cell and indicates dependence on IL-7 signals for homeostatic maintenance (45–50). We then used an unbiased least absolute shrinkage and selection operator (LASSO) regression analysis (51) to identify the cell surface markers of T cell activation expressed by each memory T cell subset *ex vivo* that best predicted the features of a population of cells within each subset that would support an inducible HIV reservoir. These models were optimized via leave-one-out cross-validation, and the model with the least cross-validated mean square error was chosen (0.37 for T_CM_ cells and 0.32 for T_EM_ cells) (Fig. S3a and b). Our data show that the induction of HIV msRNA in *ex vivo* T_CM_ cells was best associated with cells expressing high Ki67, a marker of cell proliferation, and low HLA-DR and PD-1 (Table 1, T_CM_ subset; Fig. S3a), with a cross-validated mean square error close to 0 to 0.3 and an adjusted *R*^2^ of 73%, indicating that these proliferating cells were partially activated and suggesting a response driven by γ-chain cytokines, which we have shown are critical in HIV reservoir maintenance (Table 1, T_CM_ subset) (1). Whereas in the T_EM_ cells, expression of high Ki67, PD-1, and CD127 best predicted frequencies of cells with inducible HIV msRNA (Table 1, T_EM_ subset, and Fig. S3b, with a cross-validated mean square error close to 0 to 0.3 and an adjusted *R*^2^ of 70%). These cells also express low HLA-DR and CD38, supporting previous observations that the reservoir in the T_EM_ subset is maintained in resting populations (52, 53).

**TABLE 1:** Activation markers expressed in T_CM_ and T_EM_ cells that best predicted the frequency of cells expressing inducible HIV msRNA^*a*^

Subset	Activation marker	Coefficient
T_CM_ cells	Ki67	0.5
	HLADR	–0.1
	PD-1	–0.8

T_EM_ cells	PD-1	0.03
	CD127	0.2
	Ki67	0.6
	HLADR/CD38	–0.3

a A LASSO regression model was built with the activation markers as independent variables and the frequency of cells expressing inducible HIV msRNA as the dependent variable. The models were optimized via leave-one-out cross-validation, and the least cross-validated mean square error was determined. The regression coefficient for each marker is provided.

**FIG 4:**
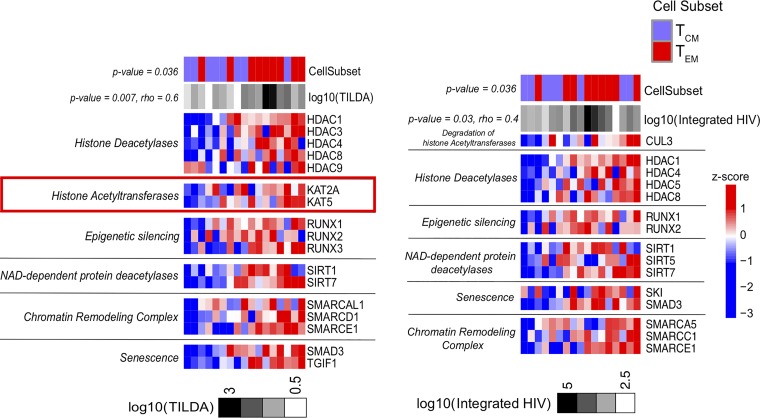
The HDAC pathway expressed in the T_EM_ subset is correlated with the induction of HIV msRNA. Heatmaps of the leading-edge genes, enriched among the genes correlated with the induction of HIV msRNA measured by TILDA (left) and with the levels of integrated HIV DNA on sorted CD4^+^ T_CM_ and T_EM_ subsets from the Florida cohort (right), by running GSEA using the HDAC pathway gene set from the Pathway Interaction Database (PID; http://pid.nci.nih.gov/) and custom gene sets of chromatin remodeling (PubMed identifier, 29236683) as the database (*P* value < 0.05). Samples were ordered by increasing the expression of the genes associated with log_10_(TILDA) or log_10_(integrated HIV DNA) (mean rank ordering). The association between the mean rank and TILDA result is then tested with a Spearman correlation, and its *P* value and rho are indicated next to the TILDA result and integrated HIV DNA annotation bar. The increasing gene expression is associated with increasing TILDA values (*P* value = 0.007, rho = 0.6) and with integrated HIV (*P* value = 0.03, rho = 0.4). A Wilcox test is then performed to assess the difference in mean ranks between T_EM_ and T_CM_ cells. The Wilcox test *P* value is then indicated next to the cell subset annotation bar, and these genes show higher expression in T_EM_ than in T_CM_ cells (*P* value = 0.036).

We assessed in sorted CD4^+^ T_CM_ and T_EM_ subsets the enrichment of pathways involved in epigenetic modifications and transcriptional control that include NAD-dependent histone deacetylase (sirtuins) (54, 55), NAD-independent histone deacetylase (56, 57), histone acetyltransferases (HATs) (58–61), and other chromatin-modifying enzymes (SWI/SNF-related, matrix-associated, actin-dependent regulator chromatin group A [SMARCA]) (62–64). Genes that control the establishment of HIV latency (HDACs) (64) as well as epigenetic silencing genes (RUNXs) and SMARCAs, i.e., genes that inhibit the unwinding of chromatin, were all expressed at higher levels in T_EM_ than in T_CM_ cells (Fig. 4). Importantly, heightened expression of these genes was positively correlated with the levels of inducible HIV msRNA as well as with frequencies of cells with integrated HIV DNA (Fig. 4a and b; Table S5). The expression of genes involved in the reactivation of latent HIV, like the HATs KAT2A and KAT5 (65), was higher in the T_EM_ subset and specifically correlated with the highest levels of inducible HIV msRNA (Fig. 4a).

Transcriptional profiling and phenotypic analyses showed that expression of genes, pathways, and cell surface markers involved in T cell activation/proliferation in the T_EM_ subset enhanced the response of this subset to LRAs compared to that of the T_CM_ or T_TM_ subset. The enhanced response of the T_EM_ subset also correlates with chromatin remodeling, such as modulation of histone acetyltransferase and deacetylase enzymes. These data, together with the phenotypic analyses, indicate that distinct mechanisms may regulate HIV latency in different memory CD4^+^ T cell subsets.

### The LARA recapitulates the complex dynamics of the establishment and maintenance of the latent reservoir in different memory T cell subsets.

**FIG 5:**
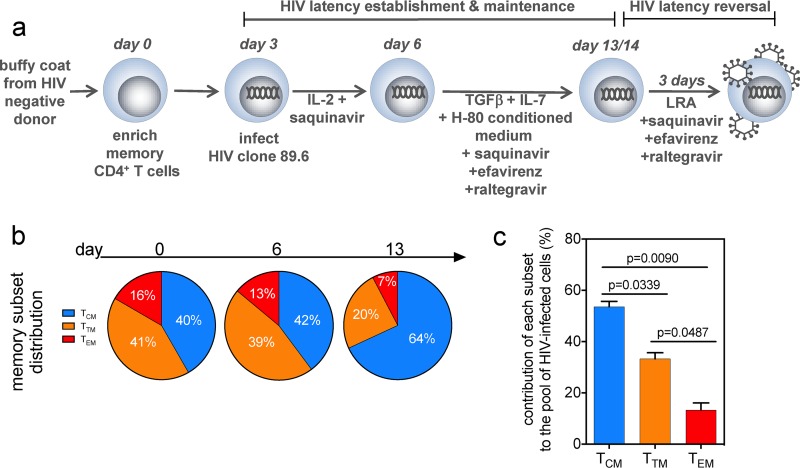
*In vitro* model of HIV latency LARA recapitulates the dynamics of HIV infection in memory CD4^+^ T cell subsets. (a) Schematic of the LARA model. On day 0, resting memory CD4^+^ T cells are enriched and allowed to rest before infection on day 3 with the full-length replication-competent HIV clone 89.6. Immediately after spinoculation, cells are resuspended in IL-2 and saquinavir. Infected cells are incubated for an additional 3 days before being introduced into latency culture conditions on day 6. Latency culture medium contains TGF-β, IL-7, conditioned medium from the H-80 cell line, and an antiretroviral cocktail of saquinavir, efavirenz, and raltegravir. After 7 days, the latently infected cells are exposed to LRAs in the presence of the triple-antiretroviral cocktail. Latency reversal is quantified by assessing percentages of CD4^−^ Gag^+^ cells by flow cytometry. (b) The memory cell subset distribution was monitored on days 0, 6, and 13 in LARA culture. The proportions of the population in the T_CM_, T_TM_, and T_EM_ subsets at each time point are indicated in the pie slices (*n* = 23). (c) The contribution of each subset to the pool of HIV-infected cells was determined. On day 13, latently infected cells generated in the LARA were sorted into T_CM_, T_TM_, and T_EM_ populations and were assessed for the presence of integrated HIV DNA by quantitative PCR. The contribution of each subset is expressed as the frequency of integrated HIV DNA by the proportion of cells present in each subset in the total population. Paired *t* test *P* values are indicated (error bars indicate SD; *n* = 3).

We developed a latency and reversion assay (LARA), an *in vitro* model, to characterize the mechanisms that trigger HIV latency and reversal in each memory CD4^+^ T cell subset and confirm our *ex vivo* findings (Fig. 5a). To establish HIV latency *in vitro*, LARA conditions were optimized to mimic the homeostatic T cell environment in lymph nodes, which supports the long-term maintenance of memory CD4^+^ T cells, including the addition of transforming growth factor β (TGF-β) and IL-7, two cytokines that promote T cell survival and quiescence (29, 66–71), and conditioned medium from the glioblastoma cell line H-80 (72, 73), which also contains cytokines that promote quiescence (TGF-β1, -2, and -3 and IL-9) and survival (IL-21) (Fig. S4a) (74). Phenotypic assessment showed that LARA conditions supported the maintenance *in vitro* of all memory subsets (T_CM_, T_TM_, and T_EM_) as well as functional CD4^+^ T cell subsets (Th1, Th2, Th17) (Fig. 5b; Fig. S4b). To determine the frequency of infected cells in each memory subset, we sorted LARA T_CM_, T_TM_, and T_EM_ cells and quantified integrated HIV DNA (26). After normalizing the frequency of integrated HIV by the proportion of cells in each subset, we demonstrated that LARA culture conditions recapitulated the distribution hierarchy of HIV-infected memory CD4^+^ T cell subsets similar to those previously observed *in vivo* (1), with the T_CM_ subset contributing the most to the frequency of cells carrying integrated HIV DNA, followed by the T_TM_ and T_EM_ subsets (Fig. 5c).

**FIG 6:**
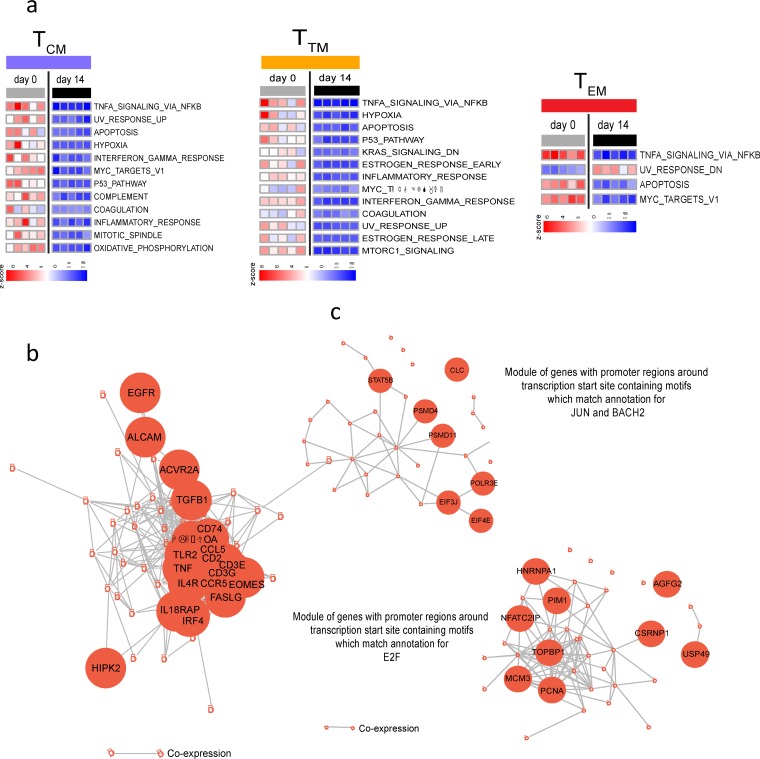
Transcriptional profiling of memory subsets from LARA cultures. (a) Pathways significantly (*P* value < 5%) enriched among the genes differentially expressed in a comparison of day 14 to day 0 results for T_CM_, T_TM_, and T_EM_ cells, respectively, by GSEA. The expression of pathways in each sample is represented by their z-scores, calculated using SLEA. The rows represent pathways, and columns represent samples. (b) GSEA of the genes differentially expressed between the T_EM_ and T_CM_ subsets in the LARA *in vitro* (day 14) culture using the Hallmark gene sets of MSigDB. Represented are the network of genes of the Hallmark allograft rejection pathway and the IL-2 STAT5 signaling pathway upregulated in effector memory cells (T_EM_) (*P* values < 5%), with edges inferred by GeneMANIA showing coexpression between genes, with certain genes of the pathways highlighted in larger nodes. (c) GSEA of the genes differentially expressed between T_EM_ and T_CM_ subsets in LARA *in vitro* culture, using the gene sets that share transcription factor binding sites defined in the C3 gene sets (TRANSFAC version 7.4) of MSigDB. The gene sets upregulated in T_EM_ cells at a *P* value of <0.05 were grouped into related modules by the enrichment map strategy. Modules were defined if gene sets had an overlap of at least 25% genes between them. The genes represented in at least 50% of gene sets of a module and the edges inferred by GeneMANIA as being coexpression between genes are represented.

Transcriptional profiling was performed to confirm that cells in LARA *in vitro* culture maintained the signatures characteristic of *ex vivo* memory subsets. Sorted memory CD4^+^ T cells that had been cultured in the presence of TGF-β and IL-7 showed transcriptional profiles similar to those of *ex vivo*-derived cells (Fig. S5a to d), with 70% to 90% of pathways not showing differences in expression between *ex vivo* and *in vitro* cells. Changes in gene signatures were observed mostly in pathways downstream of TGF-β signaling, i.e., the downregulation of inflammatory pathways (IFN-γ, inflammatory response, TNF-α signaling) and metabolic pathways (oxidative phosphorylation, targets of MYC, mTORC1, a pathway critical for effector memory T cell differentiation) (Fig. 6a; Table S6) (75, 76). Importantly, T_EM_ cells exposed to TGF-β still expressed higher levels of the transcriptional machinery involved in effector T cell function and HIV transcription and replication (EOMES, CCL5, IL-18RAP, STAT5, NFATC2) than T_CM_ cells (Fig. 6b and c). These results confirm that LARA culture conditions uniquely retain phenotypically and transcriptionally distinct memory CD4^+^ T cell subsets that allowed us in a single assay to assess LRA activity in each memory subset and differential examination of the dynamics of HIV latency reversal.

### The LARA shows that T_CM_, T_TM_, and T_EM_ subsets display different efficiencies in their response to latency reversal agents.

**FIG 7:**
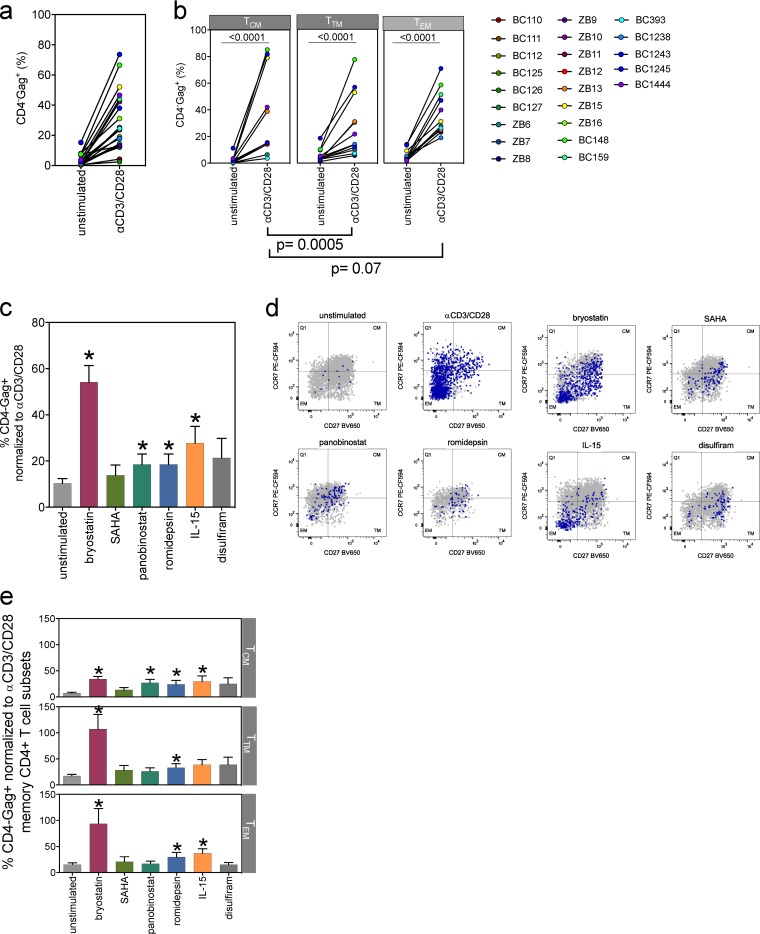
LRAs induce differential responses in different memory CD4^+^ T cell subsets. (a) Anti-CD3 (αCD3) CD28-activated cells generated in the LARA show a significant increase in the CD4^−^ Gag^+^ population over the background level (each circle represents a unique donor in individual assays; paired Wilcoxon rank sum test; *n* = 23, *P* < 0.0001). The mean of unstimulated cells is 3%, and the mean of anti-CD3- and CD28-activated cells is 28%. The median fold change in CD4^−^ Gag^+^ expression induced by anti-CD3 and CD28 antibodies is 12-fold. (b) Latency reversal was assessed in the CD4^+^ T_CM_, T_TM_, and T_EM_ subsets after TCR stimulation with anti-CD3 and CD28 antibodies (paired Wilcoxon rank sum test, *P* < 0.001 for each memory subset). The mean of the CD4^−^ Gag^+^ cell population in the T_CM_ subset negative control was 2%, the mean of the TCR-activated cell population was 33%, and the median was a 14-fold change. The mean of the CD4^−^ Gag^+^ cell population in the T_TM_ subset negative control was 6%, the mean of the TCR-activated cell population was 28%, and the median was a 4-fold change. The mean of the CD4^−^ Gag^+^ cell population in the T_EM_ negative control was 6%, the mean of the TCR-activated cell population was 37%, and the median was a 9-fold change (*n* = 12). We examined different classes of LRAs for efficiency of latency reversal in the LARA. (c) Percentages of CD4^−^ Gag^+^ cells within total memory CD4^+^ T cells after exposure to each compound were normalized to the percentage of the positive control with 1 μg/ml anti-CD3 and CD28 antibodies. LRA concentrations shown are 5 nM bryostatin (maroon bar), 1 μM SAHA (olive bar), 20 nM panobinostat (green bar), 5 nM romidepsin (blue bar), 500 ng/ml IL-15 (orange bar), and 1 μM disulfiram (dark-gray bar). *n* = 10. (Error bars indicate standard errors of the means [SEM]; asterisks indicate a *P* value of <0.05 in a comparison of the drug with the unstimulated controls, tested by a paired Wilcoxon rank sum test.) LRAs trigger different quantitative responses in memory CD4^+^ T cell subsets. (d) Representative flow plots show responses from one LARA donor. The *x* and *y* axes represent CD27 and CCR7 expression in the CD45RA^−^ memory compartment, respectively. Gray dots represent the entire memory population present in the sample overlaid with CD4^−^ Gag^+^-expressing cells (purple) to show localization within the memory subsets. Each subset is identified within the flow cytometry plot. (e) Latency reversal efficiency from LARA donors in panel c was assessed in the T_CM_ (top), T_TM_ (middle), and T_EM_ (bottom) subsets. The percentage of CD4^−^ Gag^+^ cells expressed in each subset was normalized to the signal from the positive control with anti-CD3 and CD28 antibodies (*n* = 10; error bars indicate SEM; asterisks indicate a *P* value of <0.05 for a comparison of the drug with unstimulated controls, tested by a paired Wilcoxon rank sum test).

We next quantified the frequency of cells that show latency reversal in each memory CD4^+^ T cell subset. Anti-CD3 and CD28 antibody TCR engagement was used to reactivate HIV from latently infected cells generated in the LARA and subsequently was the positive control for all LARA HIV reactivation (77–80). Activation with anti-CD3 and CD28 antibodies for 72 h resulted in a median 12-fold increase in the frequency of CD4^−^ Gag^+^ cells in the total memory CD4^+^ population (*P* < 0.0001) (Fig. 7a). Our results also show increased frequencies of CD4^−^ Gag^+^ cells upon TCR stimulation in all memory CD4^+^ T cell subsets, with T_CM_ cells showing the highest fold increase compared to the frequencies in T_TM_ cells (*P* = 0.0005) and T_EM_ cells (*P* = 0.07), with a median increase of 14-fold in T_CM_, 4-fold in T_TM_, and 9-fold in T_EM_ cells (Fig. 7b). To demonstrate the impact of TGF-β and IL-7 on HIV latency in the LARA, we omitted each cytokine alone or in combination and examined the resulting frequency of CD4^−^ Gag^+^ cells after activation with anti-CD3 and CD28 antibodies (*n* = 3) (Fig. S4c). Activation under standard LARA conditions resulted in the induction of ∼16.6% of CD4^−^ Gag^+^ cells, an average 8.5-fold increase over the percentage in the unstimulated control (Fig. S4c). Omission of both cytokines results in a very low frequency of CD4^−^ Gag^+^ cells (∼1.8%; 1.5-fold increase), while omission of either TGF-β or IL-7 results in frequencies of Gag^+^ CD4 T cells of ∼6.9% (4.4-fold increase) or ∼5.6% (1.4-fold increase), respectively.

We next quantified the response of latently infected memory CD4^+^ T cells in the LARA to *ex vivo* CD4^+^ T cells from virally suppressed HIV-infected individuals from the FL cohort using TILDA and several classes of LRAs, including bryostatin, the HDAC inhibitors (HDACi) panobinostat, romidepsin, and suberoylanilide hydroxamic acid (SAHA), the acetaldehyde dehydrogenase inhibitor disulfiram, and IL-15. We determined the frequency of infected cells in each assay and then assessed the maximal responsiveness of each participant to anti-CD3 and CD28 antibody stimulation in the LARA (*P* = 0.0187; *n* = 5) (Fig. S6a) or PMA plus ionomycin stimulation in *ex vivo* CD4^+^ T cells using TILDA (*P* = 0.0284, *n* = 4) (Fig. S6b) (27). Bryostatin (50 nM), panobinostat (20 nM), romidepsin (20 nM), and IL-15 (10 ng/ml) significantly induced latency reversal in memory CD4^+^ T cells in the LARA (*P* value < 0.05), with bryostatin showing the highest efficiency (mean, 25%) (Fig. S6c). The HDACi SAHA (0.5 μM) showed the lowest latency reversal (mean, 11%). Bryostatin showed the highest efficiency in latency reversal (Fig. S6d) (mean, 20%; *P* = 0.0419) in *ex vivo* CD4^+^ T cells from virally suppressed HIV-infected individuals. All other LRAs showed lower efficiency in reactivating HIV, including HDACi, in *ex vivo* samples (<10% of positive control), most probably a result of the lower frequency of cells with integrated provirus in these samples than in LARA samples. These data show that the LARA identified classes of LRAs with a hierarchy of latency reversal effectiveness that recapitulated TILDA results obtained with *ex vivo*-sorted subsets (Fig. 2b). These results validated the use of the LARA as a platform to predict the efficacy of LRAs to reactivate HIV in each memory subset.

**FIG 8:**
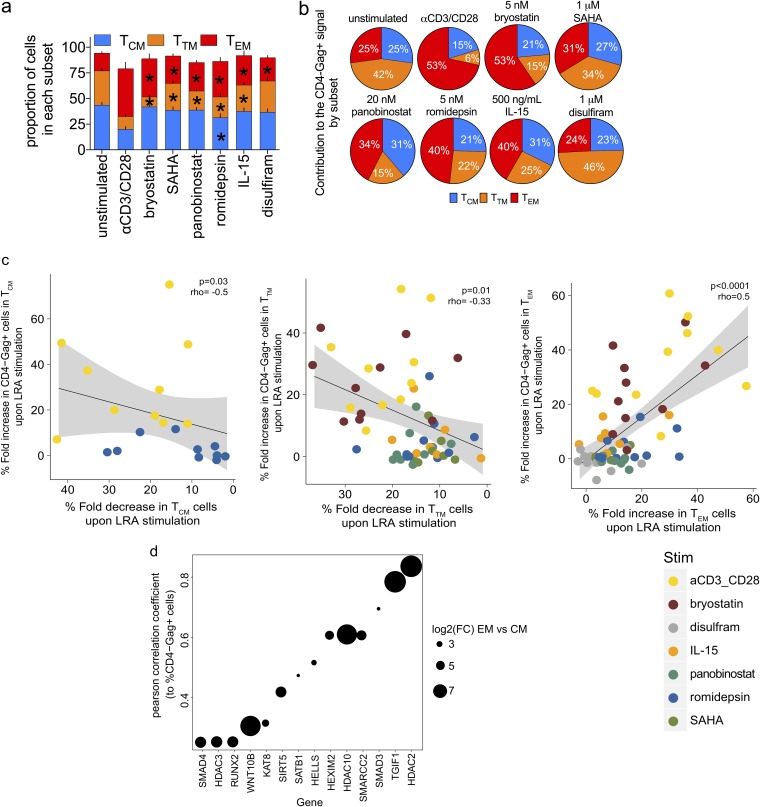
The T_EM_ subset shows the greatest contribution to latency reversal from different classes of LRAs. (a) The effect of LRAs on the distribution of the memory CD4^+^ T cell subsets was assessed and is represented by the percentage of cells in each subset in the CD45RA^−^ population (bars indicate SEM; asterisks indicate a *P* value of <0.05 in a comparison of the drug with unstimulated controls in each memory subset, tested by a paired Wilcoxon rank sum test). (b) The contribution of the T_CM_, T_TM_, and T_EM_ subsets to the overall CD4^−^ Gag^+^ signal for each LRA was determined. Each pie slice was calculated using the frequency of cells in each memory subset from the CD4^−^ Gag^+^ population. Frequency is indicated within each pie slice. *n* = 10. (c) Correlation between the percent change in CD4^−^ Gag^+^ cells after LRA stimulation and the percent change in cells in the T_EM_, T_TM_, and T_CM_ subsets after exposure to LRAs. Each circle represents an independent donor after administration of anti-CD3 CD28 (yellow), bryostatin (brown), disulfiram (gray), IL-15 (orange), panobinostat (green), romidepsin (blue), or SAHA (olive). *P* values of Spearman’s correlation test are indicated. (d) Genes of the HDAC gene sets expressed at day 14 in LARA *in vitro* culture in T_CM_ and T_EM_ cells correlated with the percentage of CD4^−^ Gag^+^ cells measured postreactivation in T_CM_ and T_EM_ cells by anti-CD3 and CD28 antibody stimulation. The enrichment of gene sets was tested by GSEA using the PID HDAC pathway gene sets and custom gene sets as the database (enrichment *P* value < 0.05). The genes are represented on the *x* axis, and the Pearson correlation coefficient of each gene to the HIV msRNA is represented on the *y* axis. The size of the dots represents the log_2_ fold change of the gene in T_EM_ cells compared to that in T_CM_ cells.

To further characterize the response to LRAs, we identified the 50% effective concentration (EC_50_) for each compound (data not shown) and assessed the responsiveness to LRAs of the total memory population as well as each memory CD4^+^ T cell subset in the LARA. Memory CD4^+^ T cells showed a range of responses to bryostatin, SAHA, panobinostat, romidepsin, IL-15, and disulfiram that resulted from the differential responses of each memory subset (Fig. 7c to e). Although latency reversal with bryostatin was significant in all subsets (*P* value < 0.05), it showed the highest efficiency in T_TM_ (mean, >100%) followed by T_EM_ (mean, 94%) and T_CM_ (mean, 34%) cells. Romidepsin also demonstrated significant latency reversal (*P* value < 0.05), but the overall efficiency for any subset was low (mean, ≤33%). The latency reversal efficiency of IL-15 was significant (*P* value < 0.05) in T_CM_ (mean, 29%) and T_EM_ (mean, 37%) cells. We showed that LRAs can induce *ex vivo* CD4^+^ T cell activation and differentiation, i.e., PMA plus ionomycin and bryostatin (Fig. 2). To determine if LRA-induced latency reversal could be attributed to the differentiation of T_CM_ cells into more differentiated subsets (81, 82), we quantified T_CM_, T_TM_, and T_EM_ cell frequencies prior to and upon exposure to LRAs (Fig. 8a). T_TM_ cell frequencies significantly decreased upon exposure to all LRAs except disulfiram, while T_EM_ cells significantly increased after exposure to all tested compounds (*P* value < 0.05) (Fig. 8a). Anti-CD3 and CD28 antibodies and romidepsin were the only conditions to significantly decrease frequencies of T_CM_ cells (*P* value < 0.05). These data suggest that after exposure to LRAs, T_TM_ cells may transition more readily to T_EM_ cells. The relative contributions of the memory CD4^+^ T cell subsets to the total CD4^−^ Gag^+^ signal were varied (Fig. 8b). Similarly to what we observed in *ex vivo* T_EM_ cells (mean, 52%) (Fig. 1d), T_EM_ cells in the LARA contributed the most to frequencies of CD4^−^ Gag^+^ cells after anti-CD3 and CD28 antibody stimulation (mean, 53%) (Fig. 8b). Indeed, T_EM_ cells, which comprised the smallest proportion of the CD4^+^ T cell population (Fig. 5b) (mean, 7%), contributed the most to the CD4^−^ Gag^+^ signal for all but two of the compounds tested, SAHA and disulfiram (Fig. 8b). In contrast, T_CM_ cells, which comprised 60% of the total population and greater than half the HIV-infected cell population (Fig. 5b), comprised only 21 to 31% of the frequency of CD4^−^ Gag^+^ cells induced from all compounds tested (Fig. 8b). These data confirm the differential responses of memory CD4^+^ T cell subsets to LRAs. Further, they show that the contribution to latency reversal in CD4^+^ T cell subsets is derived predominantly from the T_EM_ subset phenotype.

Due to the observed changes in the subset distribution after LRA exposure, we next determined whether the magnitude of these changes induced by all LRAs correlated with latency reversal in our model. We had observed that TCR stimulation, bryostatin, disulfiram, IL-15, panobinostat, romidepsin, and SAHA all led to significantly increased frequencies of T_EM_ cells after 72 h of culture (Fig. 8a). Comparison of the fold increase in numbers of T_EM_ cells after LRA exposure revealed a significant correlation with increased induction of HIV Gag expression (Fig. 8c) (*P* < 0.0001). As expected, a significant decrease in the T_CM_ and T_TM_ cell frequencies also correlated with increased HIV Gag^+^ cells upon LRA exposure (T_CM_ cell *P* = 0.03; T_TM_ cell *P* = 0.01) (Fig. 8c). Together, our data indicate that LRAs which can induce quiescent T_CM_ cells to progress into more differentiated T_EM_ cells will trigger the highest latency reversal efficiency.

### T_EM_ cells show the epigenetic machinery that is required to trigger HIV p24 in the LARA.

In Fig. 4, we showed that the expression of several members of the HDAC gene family were expressed at higher levels in *ex vivo*-isolated T_EM_ cells than in T_CM_ cells and that this upregulated expression of activators and inhibitors of epigenetic changes was correlated with increased frequencies of inducible HIV msRNA. We used transcriptional profiling to test for the enrichment (using gene set enrichment analysis [GSEA]) of the HDAC gene set in T_EM_ cells and T_CM_ cells on day 14 of the LARA and quantified their correlation with the frequency of CD4^−^ Gag^+^ cells postreactivation in the memory subsets (Fig. 8d). Increases in HDAC gene expression (e.g., HDAC2, HDAC3, HDAC10), SATB1 (known to recruit HDACs), epigenetic silencers (RUNXs), and SMARCAs (which includes a helicase that controls cell cycle entry, HELLS [83]) in addition to HATs (like KAT8) on day 14 of culture correlated with an increase in the frequency of CD4^−^ Gag^+^ cells postreactivation (normalized enrichment score > 1.5; enrichment *P* value < 0.05). Moreover, all these genes showed higher expression in T_EM_ cells than in T_CM_ cells (log_2_ fold change, T_EM_ cells versus T_CM_ cells, 3 to 7) (Fig. 8d). These results confirm that T_EM_ cells are poised to express HIV upon stimulation with LRAs as they preferentially express genes known to control latency and reactivation.

## DISCUSSION

We characterized the impact of mature T cell differentiation as defined by the memory CD4^+^ T cell subset transcriptional profile and phenotype on latency reversal using both *ex vivo* CD4^+^ T cells from virally suppressed participants and a newly developed *in vitro* model of HIV latency, the LARA. Latency reactivation is thought to occur in activated cells and/or in cells undergoing active cycling and apoptosis (84–87). Our data demonstrated, *ex vivo* and *in vitro*, that T_EM_ cells had the largest inducible HIV reservoir and the highest contribution to the latency reversal signal. Our results also showed that LRAs can trigger quiescent T_CM_ cells and T_TM_ cells to differentiate and acquire cell surface phenotypes, effector functions, and signal transduction pathways which characterize T_EM_ cells. Notably, these changes in transcriptional profiles also involved the upregulation of genes that promote HIV reactivation and replication, and these changes correlated with the induction of HIV expression. Together, these data support differentiation into an effector memory phenotype to be an effective pathway to latency reversal.

We demonstrated that T_CM_ cells, T_TM_ cells, and T_EM_ cells showed distinct responses to LRAs, which is associated with changes in biological pathways specific to each subset. In the T_CM_ cell subset, bryostatin induced downregulation of FOXO1 targets. Loss of FOXO1 expression and of its downstream transcriptional targets triggers the differentiation of T_CM_ cells to effector cells and an activated effector phenotype (88). Our results also show that T_EM_ cells express several genes that control the induction of HIV latency (HDACs, SMARCAs), as well genes which can enhance reactivation and replication of HIV. Transcriptional profiling revealed that T_EM_ cells expressed pathways that support HIV production, including the PKC activation pathway, AP-1 and E2F transcription factor target and proinflammatory pathways, and the NF-κB and nuclear factor of activated T cell (NFAT) pathways, all of which were associated with greater LRA responsiveness. Multivariate modeling of phenotypic analysis of *ex vivo* cells showed proliferation and cell surface markers of T cell activation to be the best predictors of inducible HIV msRNA reactivation. They also showed that the differentiation of T_CM_ cells and T_TM_ cells triggered by LRAs to cells that express the T_EM_ cell phenotype and transcriptional profile was associated with the magnitude of latency reversal.

We also identified that changes in pathways of chromatin remodeling are associated with the inducible HIV reservoir predominantly in the T_EM_ subset after LRA treatment in *ex vivo* CD4^+^ T cells from virally suppressed participants and that expression of these pathways predicts the efficiency of latency reversal in our *in vitro* model. The T_EM_ subset is characterized by rapid response to antigen by the upregulation of effector genes. Epigenetic modulation is a mechanism of regulating gene expression, which is controlled by chromatin remodeling pathways, such as HDACs and SMARCAs. The low expression of these pathways in highly quiescent subsets, such as T_CM_ cells, show the downregulated expression of these regulatory genes. Other studies have previously demonstrated the T_EM_ subset to have more open chromatin than the T_CM_ subset, correlating with fast kinetics of effector function responses upon activation (20). Here, we show that the gene signatures of higher HDAC expression correlate with a highly inducible reservoir, suggesting a differential mechanism of HIV latency control. HDAC inhibitors like SAHA (vorinostat) have been examined in clinical trials for HIV eradication (89, 90) but have had limited demonstrable success in facilitating reservoir clearance. Our results here confirm that approaches that modulate epigenetic pathways not limited to HDACs are a significant component of HIV latency reversal. Enhancing the induction of critical transcription factor targets in each memory CD4^+^ T cell subset in combination with epigenetic pathway changes may provide a pathway to enhance latency reversal *in vivo*. Differentiation of long-lived T_CM_ cells by LRAs into T_EM_ cells, known to have a more limited life span and higher turnover rate, may provide a novel strategy that results in the accelerated decay of the HIV reservoir.

T_EM_ cells have been shown to harbor more intact proviruses than T_CM_ cells (24), while also maintaining a higher proliferation rate and a shorter half-life (91, 92) than those of T_CM_ cells (intermitotic times, 15 days and 48 days, respectively [14]). Using TILDA on sorted memory CD4^+^ subsets, we show the capacity of the epigenetic and transcriptional status of each subset to support the LTR-driven expression of multispliced HIV RNA and that T_EM_ cells harbor the highest frequency of cells induced to express HIV RNA even after normalization to the frequency of copies of integrated provirus. These data provide evidence that T_EM_ cells provide an ideal cellular environment to support HIV replication upon activation. LASSO regression analysis identified *ex vivo* markers of each subset that correlated with the highest frequency of the measured inducible HIV reservoir after stimulation, including Ki67 in T_CM_ cells and PD-1, CD127, and Ki67 in T_EM_ cells, suggesting that the subsets expressing these markers were important in defining the frequencies of latently infected cells.

The mechanism for how T_EM_ cells maintain a higher frequency of intact provirus is not well understood, and future studies will focus on characterizing the role of the T cell phenotype in infection, establishment of latency, and HIV persistence. Interestingly, multiple studies have demonstrated T_EM_ cells to be very sensitive to cytotoxic T lymphocyte (CTL)-mediated killing (93, 94). However, elimination of cells infected with replication-competent HIV is hampered by the expression of viral proteins that mediate evasion of immune system detection (nef, env) (95–98) or induce cell cycle arrest (vpr) (99, 100), which may limit the effective clearance of infected T_EM_ cells *in vivo*.

We also present a model of HIV latency, the LARA, that allowed us to examine latency establishment, maintenance, and reversal in memory CD4^+^ T cell subsets. In this model, TGF-β, a pleiotropic mediator of broad aspects of cell development and homeostasis (101, 102) with well-characterized potent inhibitory effects on CD4^+^ T cell proliferation and differentiation (29, 66–69), was employed to induce HIV latency. A study by Barouch et al. showed in a simian immunodeficiency virus (SIV) model that triggering of the TGF-β signaling pathway in all lymphoid tissues was one of the earliest events after HIV infection that correlated strongly with viral dissemination and was concomitant with suppression of innate immunity and likely seeding of the reservoir (103). TGF-β is produced by T cell regulators (Tregs), a subset of cells known to be present in lymph nodes, a major site of HIV persistence (104). Hence, the use of TGF-β in the LARA to establish and maintain HIV latency exploits a pathway that triggers T cell quiescence *in vivo*. We provide a rationale extending the role of this pathway in the establishment and reversion of HIV latency which should lead to the development of strategies that target TGF-β to reactivate HIV from latent stores. In addition to TGF-β signaling, γ-chain family cytokine IL-7 signaling has been shown to be essential for T cell survival and homeostasis (46, 105–107) and to promote HIV persistence *in vitro* and *in vivo* (70, 71, 108, 109). The antagonistic effect of TGF-β allowed the addition of IL-7 in the LARA for the maintenance of memory CD4^+^ T cells subsets without triggering increased viral replication (110). Analysis of transcriptional profiles confirmed that memory CD4^+^ T cell subsets in the LARA maintained their phenotype after 14 days of *in vitro* culture and showed that the perturbed pathways were mostly those downstream of TGF-β. Coincidentally, T_CM_ cells show the highest enrichment in TGF-β-regulated genes and include mostly genes that inhibit cell cycle entry and T cell activation; expression of these genes by T_CM_ cells may provide a mechanism that explains their refractoriness to HIV reactivation. Together, these conditions support TGF-β as an extrinsic signal that can trigger the establishment and maintenance of latent HIV-infected T_CM_, T_TM_, and T_EM_ cells in the LARA.

Using the LARA model, we show that the increase in the number of cells with an effector memory phenotype after treatment with any LRA, including anti-CD3 CD28 cells, is significantly correlated with latency reversal. The proportion of cells transitioning within the CD4^+^ T cell population from the T_CM_ to the T_EM_ phenotype correlates with increased latency reversal. Importantly, although we do observe changes in cell surface markers specific to T_CM_ upon addition of an LRA, transcriptional profiling of these cells highlights the increased expression in these T_CM_ cells of genes that are involved in the acquisition of effector function and that support HIV replication. Significantly, previous studies have demonstrated that responses to anti-CD3 CD28 costimulation specifically result in an increase in effector function in these cells (111, 112), supporting a role for transition from quiescence to effector function in latency reversal. We also note that our *in vitro* latency model employs the protease inhibitor saquinavir immediately after memory CD4^+^ T cells are exposed to the replication-competent virus; this limits the infection to a single round and also limits the accumulation of inactivating mutations or deletions that may be observed in the *ex vivo* samples, which can influence the observed frequency of HIV latency reversal. Additionally, our positive control for the LARA system is anti-CD3 CD28 activation. T_CM_ cells express high levels of CD28 and therefore may be more responsive specifically to this mechanism of activation, which may provide a higher activation signal to the T_CM_ subset than to the other subsets.

Here, we have confirmed in two experimental systems, i.e., *ex vivo* analysis of T cell subsets and analysis of reactivation of HIV in an *in vitro* model of latency, that expression by T_EM_ cells of genes involved in T cell activation/proliferation supports a cellular environment which is more favorable to HIV replication and which can explain their enhanced response to LRAs compared to those of T_CM_ or T_TM_ cells. These data suggest that efficient LRAs need to trigger a transcriptional program that will lead to the differentiation of highly quiescent cells with an activated phenotype capable of supporting HIV replication, i.e., T_CM_ to T_EM_ cells. IL-15, a cytokine known to trigger the differentiation of T_CM_ into T_EM_ cells also was shown to induce HIV expression from latently infected cells. Our results also suggest that anti-inflammatory cytokines, including TGF-β, may be potential targets for antibodies that can neutralize their capacity to inhibit innate and adaptive immune responses and to induce HIV latency. Our data suggest that Tregs, which produce TGF-β, may also be targeted to facilitate reactivation of latent HIV. Recent reports have shown that depletion of Tregs in SIV-infected aviremic nonhuman primates restores SIV replication (104). Strategies that target anti-inflammatory cytokines, such as TGF-β, also promote memory T cell differentiation, which as our results suggest results in HIV production. Overall, our findings pave the way for novel interventions that can restore HIV production and make the virus visible to a rejuvenated immune system capable of eliminating virally infected cells.

## MATERIALS AND METHODS

### Participants and samples.

Forty-seven HIV-infected participants receiving suppressive ART were recruited at the University of California, San Francisco (UCSF). Participants received ART for >3 years and had a CD4^+^ T cell count of >350 cells/μl and an HIV RNA level of <40 copies/ml, as measured by the Abbott real-time HIV-1 PCR for at least 3 years. Whole blood (50 ml) was collected by regular blood drawing. All participants signed informed consent approved by the UCSF review board.

Twenty-two additional HIV-infected participants were recruited at the Midway Immunology Research Center (Fort Pierce, FL). All participants signed informed consent approved by the Martin Memorial Health Systems review board. None of the participants under ART experienced any detectable plasma viremia at the time of study, as assessed by viral load measurement using the AmpliPrep/Cobas TaqMan HIV-1 test (v 2.0; Roche), with a detection limit of 20 copies/ml of plasma. All four individuals had been on successful ART for >36 months. All participants underwent leukapheresis to collect large numbers of PBMCs.

### Cell culture.

Primary memory CD4^+^ T cells and H-80 cells were maintained in RPMI 1640 medium (Cellgro; 10-040-CV) supplemented with 10% fetal bovine serum (FBS; PAA; A15-752), 1% penicillin-streptomycin (Cellgro; 30-001-CI), and 1% HEPES (Gibco; 15630-080) complete cRPMI (cRPMI). H-80 feeder cells (U-251 MG; Cell Lines Service) were maintained as a monolayer to a maximum 80% confluence. 293 cells used for virus production were maintained as a monolayer in Dulbecco’s modified Eagle’s medium (DMEM; Gibco; 11995-065) plus 10% fetal bovine serum and 1% penicillin-streptomycin (cDMEM).

### Plasmid preparation.

The following reagent was obtained through the NIH AIDS Reagent Program, Division of AIDS, NIAID, NIH: p89.6 from Ronald G. Collman (113–115). p89.6 that had been transformed into Escherichia coli was grown overnight in the presence of ampicillin at 30°C, and plasmid preparations were subsequently made using a Qiagen maxiprep kit (Qiagen; 12963) according to the manufacturer’s protocol. Plasmid DNA was resuspended in elution buffer to a final concentration of 1 μg/μl.

### Virus production.

Ninety times 10^6^ low-passage-number 293 cells were seeded into 10-layer CellBIND Surface HYPERFlask M cell culture vessels (Corning; 10030) in cDMEM and allowed to adhere overnight at 37°C in 5% CO_2_. The next day, 293 cells were transfected with p89.6 DNA by using 25-kDa linear polyethylenimine (PEI; Polysciences, Inc.; 23966) as a carrier and 150 mM NaCl as a diluent. Cells were incubated with the transfection cocktail in the HYPERFlask for 1 day before the medium was exchanged for UltraCULTURE serum-free medium without l-Gln (Lonza; 12-725F) containing 1% HEPES, 1% GlutaMAX (Gibco; 35050-061), and 0.6 mg/ml glucose. The transfected 293 cells were then incubated for an additional 2 days. Viral supernatants were then collected, filtered (Sigma; Z358274-12EA filter), and treated with DNase I (Invitrogen; 18068-015) for 30 min at 37°C to remove any residual plasmid DNA before being aliquoted and stored at −80°C.

### Isolation of total CD4*^+^* T cells.

PBMCs were isolated from leukapheresis products by Ficoll-Hypaque density gradient centrifugation and cryopreserved in liquid nitrogen. After PBMC thawing, total CD4^+^ T cells were isolated from cryopreserved PBMCs by negative selection according to the manufacturer’s protocol with the EasySep human CD4^+^ T cell enrichment kit (Stemcell Technologies; 19052).

### Isolation, spinoculation, and culture of CD4*^+^* T memory lymphocytes.

For LARA culture, PBMCs were isolated by Ficoll-Hypaque density gradient centrifugation of buffy coats from HIV-negative healthy donors. PBMCs were immediately processed to enrich for memory CD4^+^ T cells by negative selection according to the manufacturer’s protocol with the EasySep human CD4 memory T cell enrichment kit (Stemcell Technologies; 10157). Purified memory CD4^+^ T cells (>98%) were allowed to rest in cRPMI at 37°C in 5% CO_2_ for 3 days at a density of 2 × 10^6^ cells/ml. Following the resting period, cells were spinoculated at 2,500 rpm for approximately 2.5 h at 30°C with 89.6, a replication-competent clinical isolate (113–115), at 100 ng/ml p24/million CD4^+^ T cells. Polybrene (Sigma; H9268) was added to a 1-μg/ml final concentration. Cells were spinoculated at 2,500 rpm for approximately 2.5 h at 30°C. After spinoculation, the viral supernatant was removed and infected cells were cultured at a density of 1 × 10^6^ cells/ml in cRPMI supplemented with 30 U/ml IL-2 (R&D Systems; 202-IL) and 5 μM saquinavir (NIH AIDS Reagent Program; 4658) at 37°C in 5% CO_2_ for 3 days. The antiretroviral drugs were added to suppress viral spread and to prevent preintegration latency. On day 6, purified CD4^+^ T memory cells were resuspended at 2 × 10^6^ cells/ml in a latency cocktail of 50% cRPMI, 50% supernatant harvested from an H-80 feeder cell line, 20 ng/ml recombinant human TGF-β1 (PeproTech; 100-21C), 40 ng/ml recombinant human IL-7 (R&D Systems; 207-IL), and an antiretroviral cocktail of 100 nM efavirenz, 200 nM raltegravir, and 5 μM saquinavir (NIH AIDS Reagent Program; 4624, 11680, and 4658). On day 10, half of the culture medium was replaced with freshly prepared latency cocktail and culture volume was adjusted to maintain the cellular density of 2 × 10^6^ cells/ml. Cells were harvested on day 13 or 14. For phenotypic and virologic analyses, cell samples were taken on days 0, 3, 6, 8, 10, and 14.

### Latency reversal with LRAs in the LARA.

On day 13 of the LARA, cells were washed, counted, and plated for latency reversal at a density of 1 × 10^6^ cells/ml in cRPMI in the presence of 100 nM efavirenz, 200 nM raltegravir, and 5 μM saquinavir and the LRA indicated. Cells were stimulated with1 μg/ml plate-bound OKT3 and 1 μg/ml soluble CD28 (BioLegend; 302933), bryostatin (Aphios; APH-09061), SAHA (a generous gift from Merck & Co., Inc., Kenilworth, NJ, USA), panobinostat (Selleckchem; LBH589), romidepsin (APExBIO; FK228), disulfiram (APExBIO; A4015), or IL-15 (R&D Systems; 247-ILB). Cells were harvested after 72 h and analyzed by flow cytometry.

### Antibody labeling of cells for flow cytometry.

Cells were incubated for 30 min at 4°C with fluorescence-labeled antibodies to surface markers in phosphate-buffered saline (PBS) plus 4% FBS. After being washed, cells were fixed in PBS plus 2% paraformaldehyde for 30 min at 4°C and then used for flow cytometry analysis. For intracellular Gag detection after surface antibody staining, cells were fixed in PBS plus 2% paraformaldehyde for 10 min at room temperature before permeabilization with 0.25% saponin. Cells were then incubated with Gag fluorophore-conjugated antibody (Beckman; 6604667) in the presence of 0.25% saponin for 30 min at room temperature. After a washing step, cells were fixed in PBS plus 2% paraformaldehyde for 30 min at 4°C. For the Treg and TH1/TH2/Tfh panels, cells were permeabilized and fixed after incubation with surface antibodies according to the manufacturer’s protocol with a Foxp3/transcription factor staining buffer set (eBioscience; 00-5523-00) to detect intracellular transcription factors. After a washing step, cells were fixed in PBS plus 2% paraformaldehyde for 30 min at 4°C. Cells from all panels were washed and resuspended in PBS plus 4% FBS prior to flow cytometric analysis.

Antibody panels used in these studies were as follows: the memory CD4^+^ T cell subset panel CD8 Pacific Blue (BD; 558207), ViViD Live/Dead aqua (Invitrogen; L34957), CD4 allophycocyanin (APC) (BD; 555349), CD27 BV650 (BioLegend; 302827), CD3 A700 (BioLegend; 300323), CD45RA APC-H7 (BD; 560674), and CCR7 phycoerythrin (PE)-Cy7 (BD; 557648). The memory subset sorting panel was as follows: CD3 fluorescein isothiocyanate (FITC) (BD; 555339), CD8 Pacific Blue (BD; 558207), CD4 APC (BD; 555349), CD45RA APC-H7 (BD; 560674), CD45RO peridinin chlorophyll protein (PerCP)-eFluor710 (eBioscience; 46-0457-42), CCR7 PE-Cy7 (BD; 557648), CD27 BV650 (BioLegend; 302827), and ViViD Live/Dead aqua (Invitrogen; L34957). For the cell cycle, we used the following: CD4 Qdot605 (Invitrogen; Q10008), CD3 PE-Cy7 (BD; 557851), CD45RA APC-H7 (BD; 560674), CD27 Qdot655 (Invitrogen; Q10066), CCR7 PE-CF594 (BD; 562381), PD-1 Alexa Fluor 647 (BD; 560838), 7-aminoactinomycin D (7-AAD) (BioLegend 420403), and Ki67 FITC (BD; 556026). For markers of activation and the proliferation panel, we used CD3 A700 (BD; 557943), CD4 Qdot605 (Invitrogen; Q10008), CD8 Pacific Blue (BD; 558207), CD45RA APC-H7 (BD; 560674), CD27 BV650 (BioLegend; 302828), CCR7 PE-Cy7 (BD; 557648), CD14 V500 (BD; 561391), CD19 V500 (BD; 561121), ViViD Live/Dead aqua (Invitrogen; L34957), HLA-DR PerCP (BD; 340690), CD38 PE (BD; 555460), CD127 PECF594 (BD; 562397), and Ki67 FITC (BD; 556026). For effector subset profiling, we used the following Treg panel: CD3 AF700 (BD; 557943), CD4 BV650 (BioLegend; 317435), ViViD Live/Dead aqua (Invitrogen; L34957), CD45RA APC-eFluor780 (eBioscience; 47-0458-42), CD25 PE-CF594 (BD; 562403), CD31 PE (BD; 560983), CD39 BV421 (BioLegend; 328213), CD127 AF647 (BioLegend; 351318), and FOXP3 AF488 (BioLegend; 320212). For the TH1/TH2/Tfh panel, we used CD3 AF700 (BD, 557943), CD4 Qdot605 (Invitrogen; Q10008), CD27 BV650 (BioLegend; 302828), CCR7 FITC (BD; 561675), CD45RA APC-H7 (BD; 560674), ViViD Live/Dead aqua (Invitrogen; L34957), CCR6 BV421 (BD; 562515), CXCR3 PE-Cy5 (BD; 561731), CXCR5 AF647 (BD; 558113), CCR4 PE-Cy7 (BD; 561034), T-bet PerCP-Cy5.5 (BioLegend; 644805), and GATA3 PE (BD; 560074).

### Flow cytometry.

Multicolor flow analysis of cell surface and intracellular marker expression was performed with a BD LSRII flow cytometer. Between 30,000 and 50,000 events were acquired for each sample using the live-cell gate. The data were analyzed with the FlowJo program.

### Cell sorting.

Central, transitional, or effector memory CD4^+^ T cells were sorted on an Aria fluorescence-activated cell sorter (FACS) (Becton, Dickinson). Subset purity was confirmed by flow cytometry analysis on the Aria immediately postsort.

### Integrated/total/2-LTR HIV-DNA.

Cell samples from HIV-infected participants and from multiple time points in the LARA were used to assess the frequencies of total, integrated, and 2-LTR circle forms of HIV DNA as previously described (26).

### Quantification of the inducible HIV reservoir.

The size of the inducible HIV reservoir was measured through quantification of HIV msRNA expression after treatment with 100 ng/ml PMA and 1 μg/ml ionomycin or 50 nM bryostatin using TILDA as previously described (27).

### Preprocessing of RNA-Seq data.

Transcriptome sequencing (RNA-Seq) was performed on samples generated in the LARA from *ex vivo* (day 0) and *in vitro* culture (day 14) cells sorted on memory CD4^+^ T_CM_, T_EM_, and T_TM_ subsets. RNA-Seq was also performed on memory CD4^+^ T cell T_CM_, T_EM_, and T_TM_ subsets from four virally suppressed HIV-infected individuals unstimulated (negative control) or stimulated with 100 ng/ml PMA and 1 μg/ml ionomycin, 10 ng/ml IL-15, or 50 nM bryostatin for 24 h. A paired-end 50-bp run was performed on an Illumina HiSeq 2500 platform, using the TruSeq kit. An average of 9 million reads per sample was generated.

An automated pipeline developed in-house that integrates online open-source tools for processing and analyzing RNA-Seq data was used to perform processing of the sequencing data, followed by downstream analysis using R-Bioconductor packages. The sequencing raw reads were trimmed off adapter sequence contaminants using Trimmomatic (116) and mapped into the Ensembl version of the human genome (Ensembl Grch38) (117) using the STAR aligner (118). The transcript abundance was then estimated by counting the number of reads mapping to the exons that are unique to the transcripts of a gene by using HTSeq (119). The obtained transcript counts were then normalized by trimmed mean of M values (TMM) analysis, with correction for the library size (120).

### Differential gene expression analysis.

The differences in transcriptomic profiles between the groups to be compared were determined by fitting a generalized linear model (GLM) (121) to each transcript of the expression data, with gene expression as a dependent variable and the group as an independent variable, followed by a likelihood ratio test for the coefficients in the linear model to test whether fold changes were different from zero. These tests were conducted using the R-Bioconductor package edgeR (122). The *P* values were corrected for multiple comparisons using the Benjamin and Hochberg (BH) method (123).

Fisher’s exact test was used to assess significance of overlap of genes differentially expressed between the *ex vivo* and the LARA day 14 culture memory subsets. Hierarchical clustering was performed on the overlapping genes to visualize the level of similarity between the different groups, using Euclidean distance as the distance measure and complete linkage clustering as the clustering method.

### Pathway enrichment analysis.

Gene set enrichment analysis (GSEA) (124) preranked with 1,000 permutations using the Hallmark gene sets (125), transcription factor targets (C3) from Molecular Signatures Database version 5.0 (MSigDB), was performed on the genes differentially expressed between comparisons. The differentially expressed genes were preranked by the decreasing order of their –log(*P* value) times their sign(log fold change) or sign(correlation coefficient) to identify pathways upregulated and downregulated or pathways correlated with HIV msRNA measured by TILDA. The obtained *P* values of the pathways were corrected for multiple comparisons using the BH method. Pathways at a nominal and adjusted *P* value of <0.05 were considered significant.

For the HDAC gene sets, we used the Pathway Interaction Database (PID) HDAC class I/II/III gene sets, and we built a custom gene set that included known (from the literature) histone deacetylases, histone acetyltransferases, and other chromatin-modifying/modeling enzymes (PubMed identifier, 29236683).

Sample-level enrichment analysis (SLEA) (126) was used to represent the expression of pathways by calculating the z-score of the pathway per sample. The mean expression value of genes of the significant gene sets was compared to the mean expression of 1,000 random gene sets of the same size as the selected gene set for every sample. The difference between the observed and expected mean expression values for each gene set was calculated.

### Statistical analysis.

A paired Wilcoxon rank sum test was performed to compare paired samples, and the obtained *P* values were corrected for multiple comparisons using the BH method. All *P* values at a nominal and adjusted *P* value of <0.05 were considered significant.

### Multivariate model of activation markers that best predict the frequency of cells expressing inducible HIV msRNA.

Feature selection was performed using the least absolute shrinkage and selection operator (LASSO) (51) technique, in order to determine the activation markers expressed by memory subsets that best predicted the induction of HIV msRNA. The model was optimized using leave-one-out cross-validation, and the least cross-validated mean square error (MSE) was determined.

### Data availability.

All materials are available upon request from the authors, subject to completion of a material transfer agreement.

## Supplementary Material

Supplemental file 1

Supplemental file 2

Supplemental file 3

Supplemental file 4

Supplemental file 5

Supplemental file 6

Supplemental file 7
