# Supplementary material for: Differentiation into an Effector Memory Phenotype Potentiates HIV-1 Latency Reversal in CD4+ T Cells
Source: J Virol. 2019 Nov 26;93(24):e00969-19. doi: 10.1128/JVI.00969-19 (PMC6880164; doi:10.1128/JVI.00969-19)
Supplement: Supplemental file 1 [file JVI.00969-19-s0007.pdf]

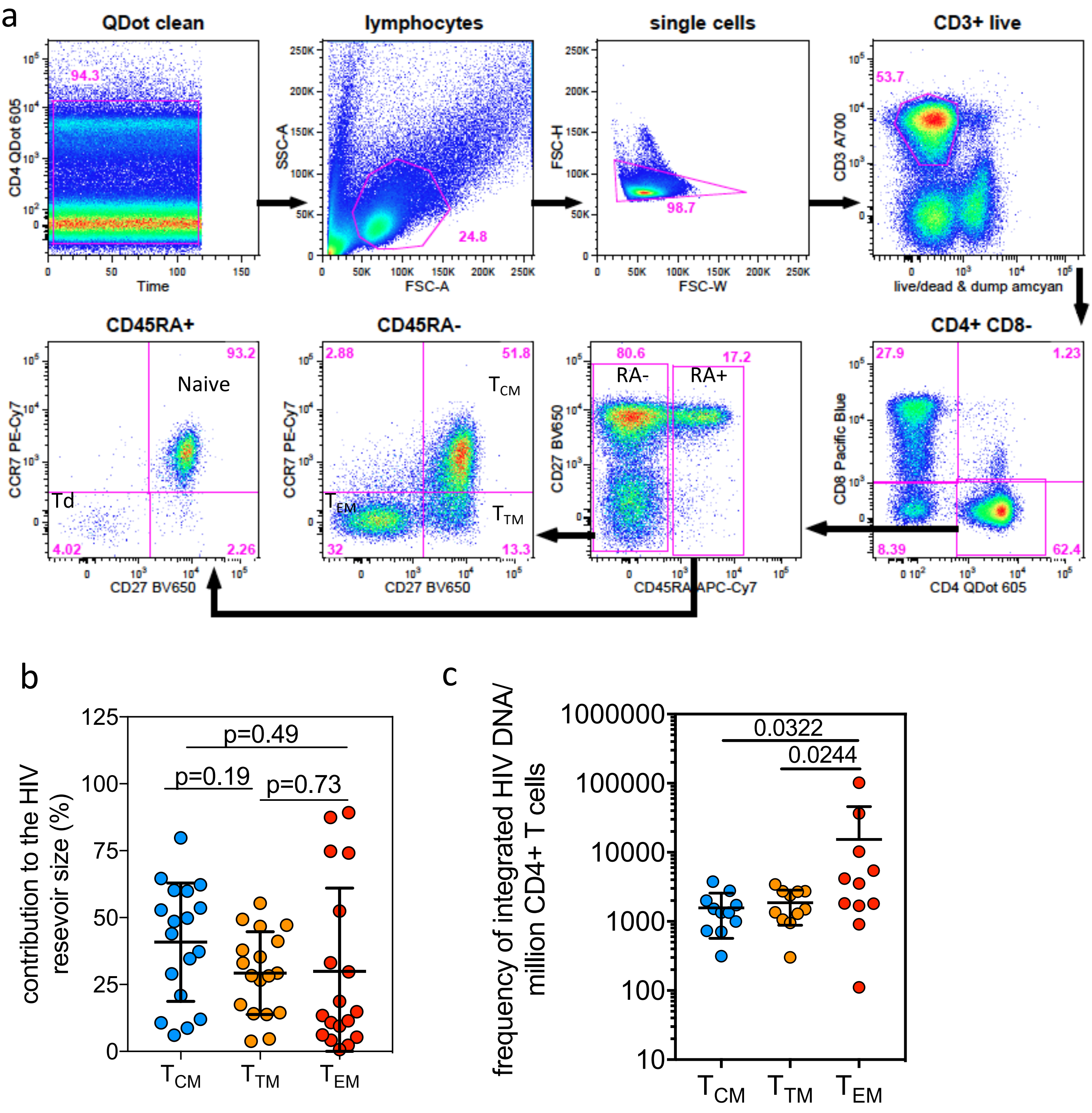

Supplementary Figure 1 (a) Representative gating strategy used in the manuscript for identifying CD4+ T cell memory subsets. **(b)** The contribution of each memory CD4+ T cell subset to the reservoir size is indicated. CD4+ T cells from virally suppressed individuals were sorted into T_{CM}, T_{TM} and T_{EM} subsets, and integrated HIV DNA was quantified (Fig 1b). The contribution of each subset is expressed as the frequency of integrated HIV DNA by the proportion of cells present in each subset in the total population. p values are indicated (each circle represents a unique participant; Wilcoxon rank sum test; n=18). **(c)** CD4+ T cells from virally suppressed individuals were sorted into T_{CM}, T_{TM} and T_{EM} subsets using the gating strategy in Supplemental Fig. 1a, and integrated HIV DNA in each subset was quantified. (Bars indicate mean with SD, Wilcoxon matched-pairs signed rank test, p values are indicated. n=11.).

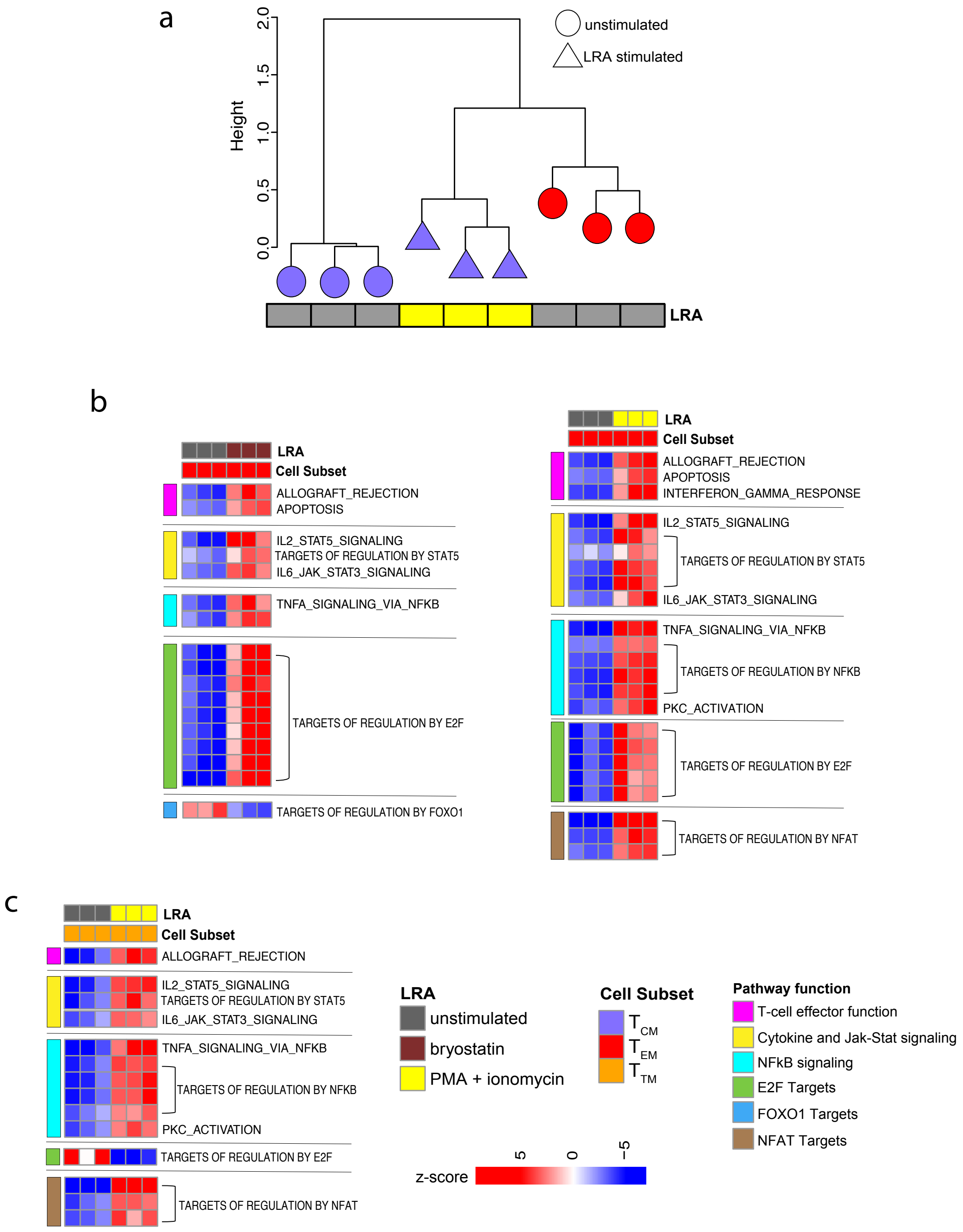

Supplementary Figure 2. (a) Hierarchical clustering on the pathways differentially expressed in the T_{CM} subset upon exposure to PMA plus ionomycin compared to their unstimulated controls (at p-value < 0.05). The pathway expression was assessed in the T_{CM} subset and in the unstimulated T_{EM} subset **(b)** Heatmap of the pathways differentially expressed upon exposure to LRAs (bryostatin and PMA plus ionomycin) compared to unstimulated controls in the T_{EM} subset (p-value < 0.05). Rows represent the pathway and columns represent samples. The color gradient represents the z-score of the pathway per sample calculated by SLEA. (**c)** Heatmap of the pathways differentially expressed upon exposure to PMA and ionomycin, compared to unstimulated controls in the T_{TM} subset (p-value < 0.05). Rows represent the pathway and columns represent samples. The color gradient represents the z-score of the pathway per sample calculated by SLEA.

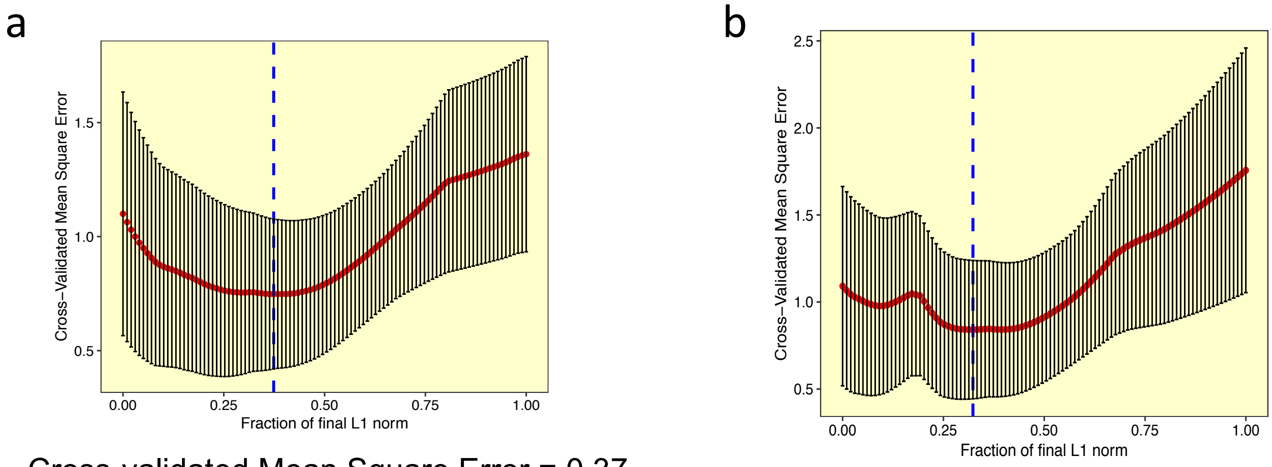

Cross-validated Mean Square Error = 0.37

Cross-validated Mean Square Error = 0.32

Supplementary Figure 3 (a/b) The cross validated mean square error plot obtained by optimization of the LASSO regression model that tested the combination of activation markers (as independent variables) that best predicted the frequency of cells expressing inducible msHIV RNA (as the dependent variable) in T_{CM} (a) and T_{EM} (b) respectively. The blue dotted line indicates the least cross-validated mean square error (0.37 for T_{CM}, and 0.32 for T_{EM}) for the models.

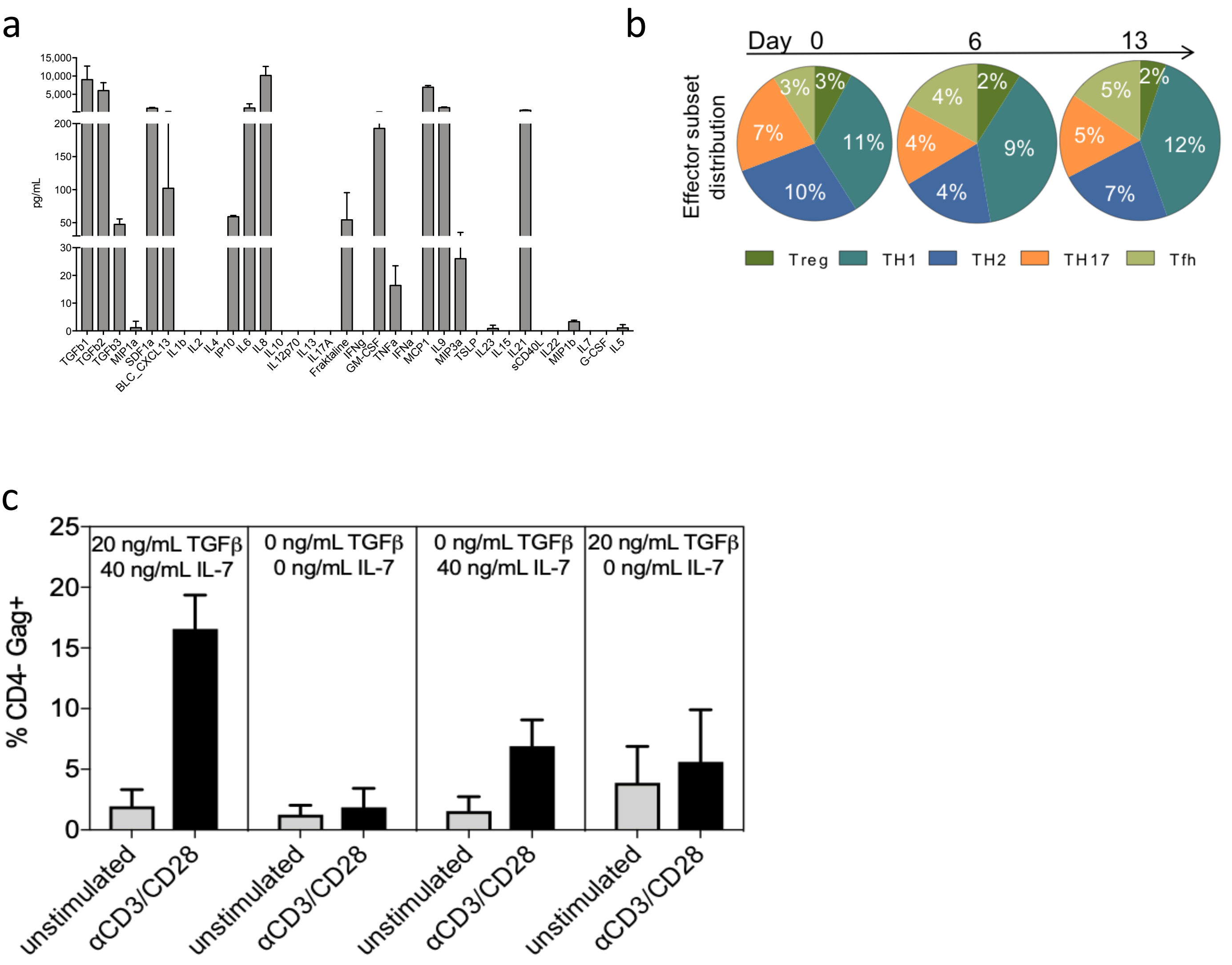

Supplementary Figure 4 (a) Conditioned medium from the H-80 cell line was analyzed using Luminex for markers of immune regulation. The pg/mL concentration of each cytokine is indicated by bars, error bars indicate SD. **(b)**. The T_{EM} subset distribution was monitored on day 0, 6 and 13 in LARA culture. The proportion of the population in Treg (olive), TH1 (teal), TH2 (aqua), TH17 (orange), and Tfh (green) subsets are indicated in each pie slice; n=5. (**c**) Cytokine omission experiments in LARA. The induction of latency reversal (%CD4-Gag+ cells) in 20 ng/mL TGF-β and 40 ng/mL IL-7 standard LARA conditions are compared to 0 ng/mL TGF-β / 0 ng/mL IL-7, 0 ng/mL TGF-β plus 40 ng/mL IL-7 or 20 ng/mL TGF-β plus 0 ng/mL IL-7. On day 13, cells were activated with αCD3/CD28 (black bars) or left untreated (grey bars) in the presence of ARVs and then monitored for the frequency of %CD4-Gag+ cells (n=3).

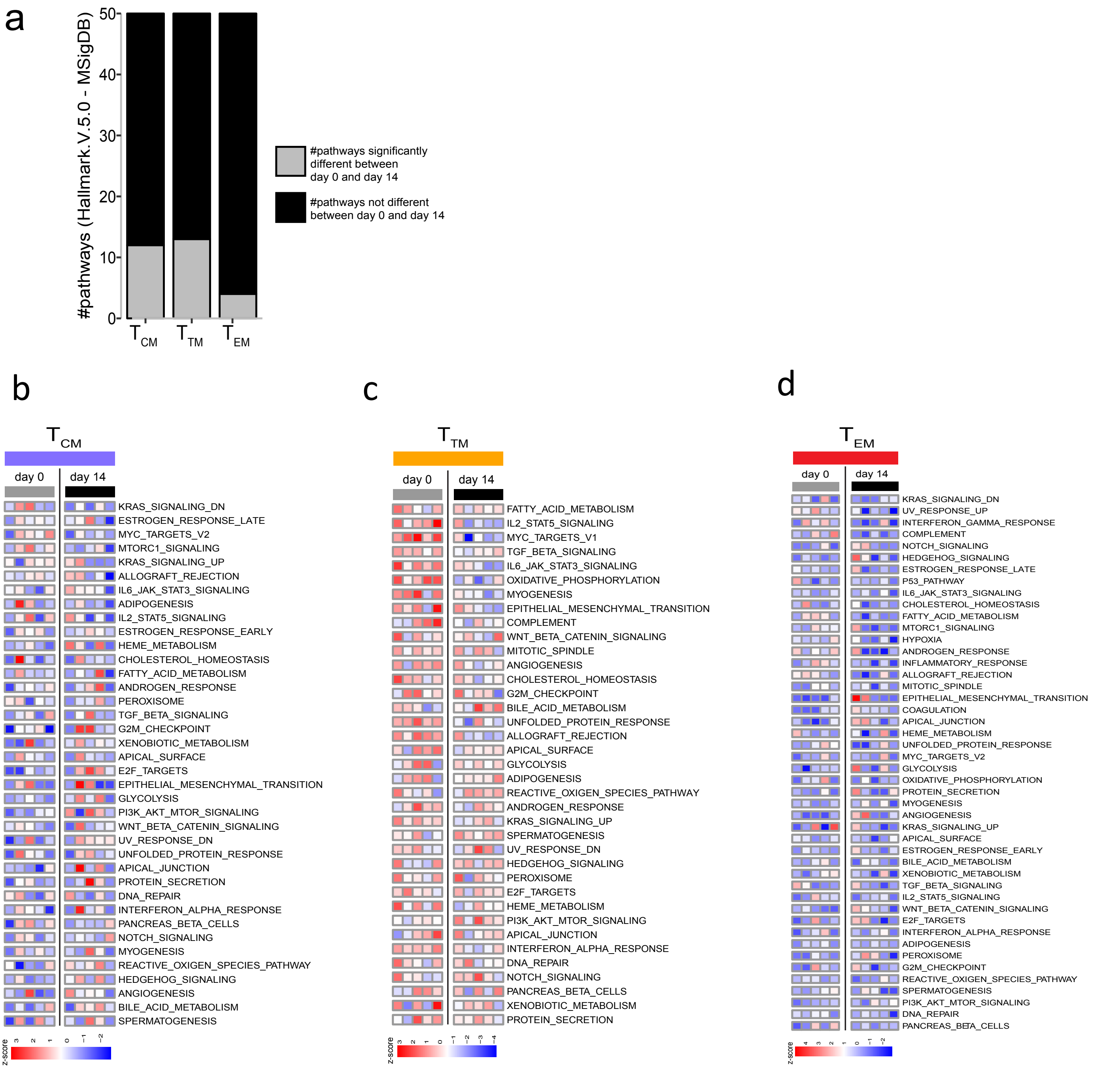

Supplementary Figure 5 (a) Determining the differential expressed genes (DEGs) between day 0 and day 14 culture conditions in each of the memory subsets, and identifying the pathways enriched among the DEGs. Stacked bar plot of the number of pathways showing significant changes (p-value < 5%) in expression between day 0 and day 14 (grey proportion), and the number of pathways that do not show significant changes in expression between day 0 and day 14 (black proportion) in each memory subset. Y-axis represents the number of Genesets / pathways in the Hallmark pathway database. 76% of pathways were similar in their expression when comparing day 0 and day 14 culture in T_{CM}, 74% in T_{TM} and 92% in T_{EM} **(b-d)** Heatmap of the pathways that do not show significant differences (p-value > 5%) between LARA *in vitro* conditions and CD4+ T cell *ex vivo* subsets in T_{CM}, T_{TM} and T_{EM} respectively. Expression of each pathway is represented by their z-score calculated using SLEA. Rows represent pathways and columns represent samples.

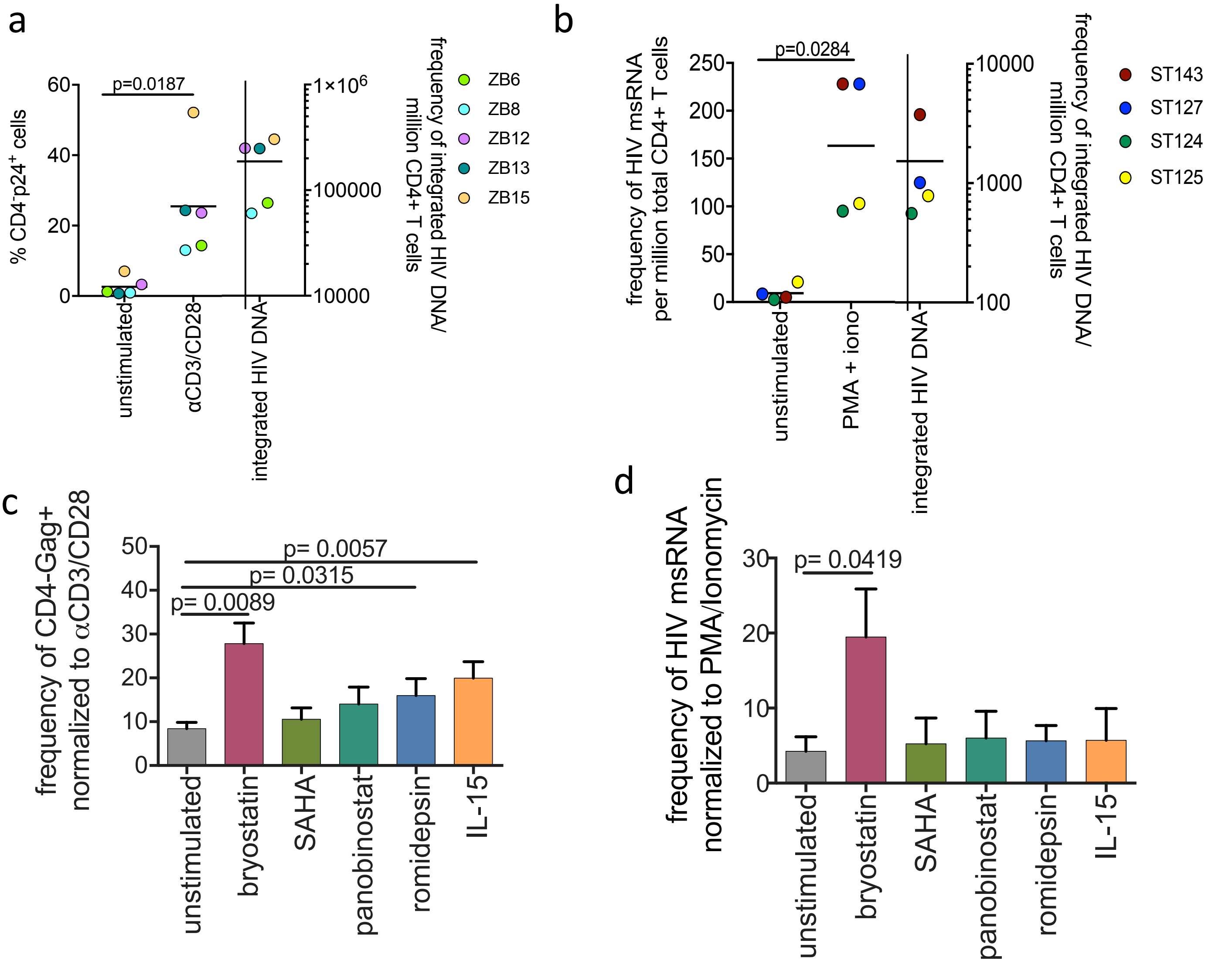

Supplementary Figure 6 (a) On day 13 of LARA, the frequency of integrated HIV DNA was determined prior to viral reactivation (right axis; n=5; each colored circle represents a unique donor in LARA). Subsequently, infected cells were cultured for 72 hours with αCD3/CD28 antibodies or left unstimulated, both in the presence of anti-retroviral compounds and the frequency of %CD4-p24+ cells was quantified by flow cytometry (left axis; p values from paired t test are indicated). **(b)** The frequency of integrated HIV DNA from *ex vivo* CD4+ T cells was determined prior to TILDA (right axis; n=4; each colored circle represents a unique participant). TILDA positive control (PMA plus ionomycin) compared to the negative control (unstimulated) is shown (left axis; p values from paired t test are indicated). **(c)** We examined the responsiveness of latently infected cells generated in LARA to different classes of anti-latency compounds using commonly reported concentrations. LRA concentrations shown in the figure- 50 nM bryostatin (maroon bar), 500 nM SAHA (olive bar), 20 nM panobinostat (green bar), 20 nM romidepsin (blue bar), 100 ng/mL IL-15 (orange bar). CD4-Gag+ signal from each LRA was normalized to the positive control 1 μg/mL αCD3/CD28. P values are shown, error bars indicate SD; n=5 independent LARA donors. **(d)** Induction of multi-spliced HIV RNA virally suppressed HIV-infected individuals using the same compounds at the same concentrations as in (a) was assessed using TILDA(27). This assay measures the frequency of cells producing tat/rev multiply spliced HIV RNA using quantitative RT-PCR. Frequency of multi-spliced HIV RNA induction was normalized to the positive control for TILDA (100 ng/mL PMA and 1 μg/mL ionomycin). P values are shown, error bars denote SD, n=4 independent participants from Table 1.
